# Organellar genome analysis reveals endosymbiotic gene transfers in tomato

**DOI:** 10.1371/journal.pone.0202279

**Published:** 2018-09-05

**Authors:** Hyoung Tae Kim, Je Min Lee

**Affiliations:** Department of Horticultural Science, Kyungpook National University, Daegu, Korea; Institut Pasteur, FRANCE

## Abstract

We assembled three complete mitochondrial genomes (mitogenomes), two of *Solanum lycopersicum* and one of *Solanum pennellii*, and analyzed their intra- and interspecific variations. The mitogenomes were 423,596–446,257 bp in length. Despite numerous rearrangements between the *S*. *lycopersicum* and *S*. *pennellii* mitogenomes, over 97% of the mitogenomes were similar to each other. These mitogenomes were compared with plastid and nuclear genomes to investigate genetic material transfers among DNA-containing organelles in tomato. In all mitogenomes, 9,598 bp of plastome sequences were found. Numerous nuclear copies of mitochondrial DNA (NUMTs) and plastid DNA (NUPTs) were observed in the *S*. *lycopersicum* and *S*. *pennellii* nuclear genomes. Several long organellar DNA fragments were tightly clustered in the nuclear genome; however, the NUMT and NUPT locations differed between the two species. Our results demonstrate the recent occurrence of frequent endosymbiotic gene transfers in tomato genomes.

## Introduction

The plant cell organelles, the plastid and mitochondrion, are known to have originated from prokaryotes via endosymbiosis, and it is possible that the origin of the mitochondrion was contemporaneous with that of the eukaryotic cell, because there is no evidence of an amitochondriate phase in eukaryotic evolution [1]. Although both organelles exist together in the plant cell, the evolutionary histories of the two organellar genomes in land plants differ slightly. Plastid genomes (plastomes) from bryophytes to angiosperms are normally 120–170 kb in length [2–5], excluding certain contracted or expanded genomes [6, 7]. They are highly conserved in terms of gene content and arrangement, which is typically circular [4]. Mitochondrial genomes (mitogenomes) in land plants are more complex than plastomes. The moss mitogenome is approximately 100 kb long, and its structure has been constant for 350 My [8]. However, seed-plant mitogenomes changed rapidly [9–11]. Ribosomal protein genes and *sdh* genes were frequently lost in angiosperm mitogenomes during evolution, and are thought to have been transferred to the nuclear genome [12, 13]. Large [10, 14] and small [15–17] repeated sequences increased the size of mitogenomes in seed plants and changed their structure via reversible and non-reversible recombination, respectively [15]. Horizontal gene transfers of mitogenome sequences have been frequently observed in terrestrial plant species [18]; consequently, mitogenomes in land plants vary between 100 kb [8] and 11.3 Mb long [19]. In addition, certain plant species contain multichromosomal mitogenomes [19, 20].

Six types of gene transfer have been observed among three genome-containing organelles in plants [21]: from the plastid to the nucleus [22–24], from the mitochondrion to the nucleus and vice versa [25–27], and from the plastid to the mitochondrion [28–35]. Gene transfer from mitochondria to plastids has been reported recently [36–40], but gene transfer from the nucleus to plastids appears to rarely occur [21]. Nuclear copies of mitochondrial DNA (NUMTs) as a result of endosymbiotic gene transfer (EGT) have been widely found from protists to animals [26]. Certain NUMTs are associated with human diseases [41] and make DNA barcoding and phylogenetic analysis using mitogenomes difficult [42, 43]. Data from 85 genomes of protists, fungi, plants, and animals have revealed a correlation between genome size and the total number of NUMTs, and eukaryotes that have only one mitochondrion contain fewer NUMTs than those that have multiple mitochondria [26]. Less than 0.1% of the nuclear genomes of mammals, insects, yeasts, and some plants contain NUMTs [26], but NUMTs in *Oryza sativa* and *Arabidopsis thaliana* account for 0.1–0.2% of their nuclear genomes [44]. The integration of mitochondrial segments into the nuclear chromosome occurs by NUMTs being inserted into double-strand breaks by non-homologous end-joining machinery [45].

Similarly, nuclear copies of plastid DNA (NUPTs) have also been found in many organisms, including land plants, algae, apicomplexans, and haplophytes. The cumulative lengths of NUPTs in polyplastidic organisms are greater than those in monoplastidic organisms, except for certain species of green algae and apicomplexans [46]. However, few comprehensive studies of gene transfers among the three genomes have been conducted, because few complete land plant nuclear genomic sequences are available.

*Solanum* is one of the most economically important plant genera because it includes many valuable crops, such as the tomato, potato, and chili pepper [47]. These species are used as plant models, and their complete nuclear genomes provide insights into many aspects of plant biology [48–50]. The organellar genomes of *Solanum* have also been studied, and the plastomes of 15 *Solanum* species have been sequenced [51–57]. These complete plastome sequences increase our understanding of the evolution and phylogenetic relationships of *Solanum* species. In contrast to the plastome, complete mitogenome sequences of *Solanum* have not been completely analyzed. The first physical map of the tomato mitogenome was constructed for a male-sterile tomato that was generated via cell fusion between the tomato and potato [58]; subsequently, draft mitogenome sequences of the tomato and potato, containing numerous gaps and unordered contigs, have been generated [49] (http://www.mitochondrialgenome.org/). Therefore, if tomato mitogenome sequences are available, it would be useful to investigate EGTs among the three DNA-containing organelles because of the availability of two sets of complete nuclear genome sequences of *S*. *lycopersicum* ‘Heinz1706’ and *S*. *pennellii* ‘LA0716’ [48, 54] and plastome sequences, and the expectation of more frequent nuclear copies of organellar DNA than those of previously studied land plants owing to their larger genome sizes [26, 46].

In this study, we assembled three complete mitogenomes (two of *S*. *lycopersicum* and one of *S*. *pennellii*) and analyzed their intra- and interspecific variations. In addition, EGTs among three genomes, including two organellar genomes and the nuclear genome, were comprehensively investigated.

## Materials and methods

### Assembly and confirmation of complete mitogenome and plastome sequences

Paired-end sequencing data [59] for *S*. *lycopersicum* ‘LA1479’ (SRA accession number: ERR418122), *S*. *lycopersicum* ‘LA1421’ (SRA accession number: ERR418120), and *S*. *pennellii* ‘LA0716’ (SRA accession number: ERR418107) were obtained using the Illumina HiSeq 2000 system. Both ends of the reads were trimmed using Geneious [60] with an error probability of 0.01, and only paired-end reads longer than 50 bp were extracted. The mitogenomes were assembled using previously developed strategies, a baiting and iterations [61, 62]. Firstly, reads were mapped to the Solanaceae mitogenomes (S1 Table). The assembled reads on the reference sequences were distributed at genes excluding noncoding regions. Secondly, mapped reads were assembled *de novo* with zero mismatch and gap to generate reference contigs, before we annotated the reference contigs using Geneious [60] to confirm whether all of the ribosomal RNAs (rRNAs), transfer RNAs (tRNAs), and protein-coding regions included in the other Solanaceae mitogenomes were included in these contigs. Thirdly, reads were realigned with the reference contigs with zero mismatch and gap among reads. Consensus sequences of these mapping reads were used as new, extended reference contigs. Subsequently, reads were iteratively mapped to the new extended reference contigs generated in the previous iteration. Contig length increased in each iteration, and few of the contigs overlapped with each other. Finally, the sequence of one circular mitogenome was obtained using each raw dataset; however, the coverage depth for certain regions was higher than that for other mitogenome regions. The sequences of these high-depth regions were almost identical to the tomato plastome sequence. We designed primer sets based on these regions, and showed that these regions belonged to the mitogenome using the genomic DNA of *S*. *pennellii* ‘LA0716’. To verify the coverage depths of the mitogenomes and plastomes of the three tomato genomes, raw reads were mapped to six mitogenome and plastome sequences using the Burrows-Wheeler alignment tool (S1 Fig and S2 Table) [63].

In addition, three plastome sequences from each raw dataset were assembled to identify EGTs among the three tomato genomes. The plastome assembly strategy followed that of Kim et al. [64].

### Annotation of genes and repeat regions

All of the genes in the three tomato mitogenome sequences were annotated and compared with other mitogenome sequences of Solanaceae using Geneious [60], and protein-coding and tRNA genes were re-examined using blastp [65] and tRNAscan-SE [66], respectively. Open reading frames (ORFs) with a minimum length of 303 bp and the start codon “ATG” were annotated using Geneious [60].

Duplicated regions with a minimum repeat length of 100 bp and zero maximum mismatch were identified using Geneious [60], and 56 mitogenome sequences of core eudicots (ftp://ftp.ncbi.nlm.nih.gov/genomes/refseq/mitochondrion) were downloaded to investigate the relationship between repeat region length and total mitogenome length (S1 Table).

### Analysis of the structural evolution of tomato mitogenomes

To analyze the structural evolution of tomato mitogenomes, the three tomato mitogenome sequences were compared using Circoletto [67] and blastn with an e-value of <1 x 10^−10^ [65]. Syntenic blocks that were longer than 1 kb and contained at least one gene are summarized in S3 Table.

### Gene transfer among the three tomato genomes

Whole-genome sequences of *S*. *lycopersicum* ‘Heinz1706’ (version SL2.50) [48] and *S*. *pennellii* ‘LA0716’ (version SPENNV200) [54] were used to investigate gene transfers among the three genomes (nuclear genome, plastome, and mitogenome). Sequences of the three genomes of *S*. *lycopersicum* and *S*. *pennellii* were compared using blastn with a word size of 11, an e-value of <1 x 10^−5^, and 50,000 maximum hits. Multiple hits for the same nuclear genomic locus caused by repetitive regions of the query sequence (duplicated regions in organellar genomes) were eliminated to avoid overestimating the migration of nuclear copies of organellar DNA [46]. Nevertheless, integration of organellar DNA into the nuclear genome can be overestimated, because nuclear organellar copies could have become fragmented during evolution.

### Origin of nuclear-transferred organellar DNA

NUPTs and NUMTs that were over 3,000 bp long from *S*. *pennellii* ‘LA0716’ were used to investigate 1) whether tightly clustered organelle copies in the nuclear genome originated from a single, large original sequence of nuclear-transferred organellar DNA and 2) whether they degenerated during evolution in hotspot regions of the nuclear genome [22]. Among the regions selected, we extracted those containing more than two large organellar DNA fragments and compared them with their equivalent organellar DNA.

### Statistical analysis and graphics

All of the statistical analyses were performed using R v3.3.3 [68], and most of the figures were generated using ggplot2 [69], gridExtra [70], and genoPlotR [71] in R, excluding the mitogenome maps, which were generated using OGDRAW [72].

## Results

### Structure of tomato mitogenomes

**Fig 1:**
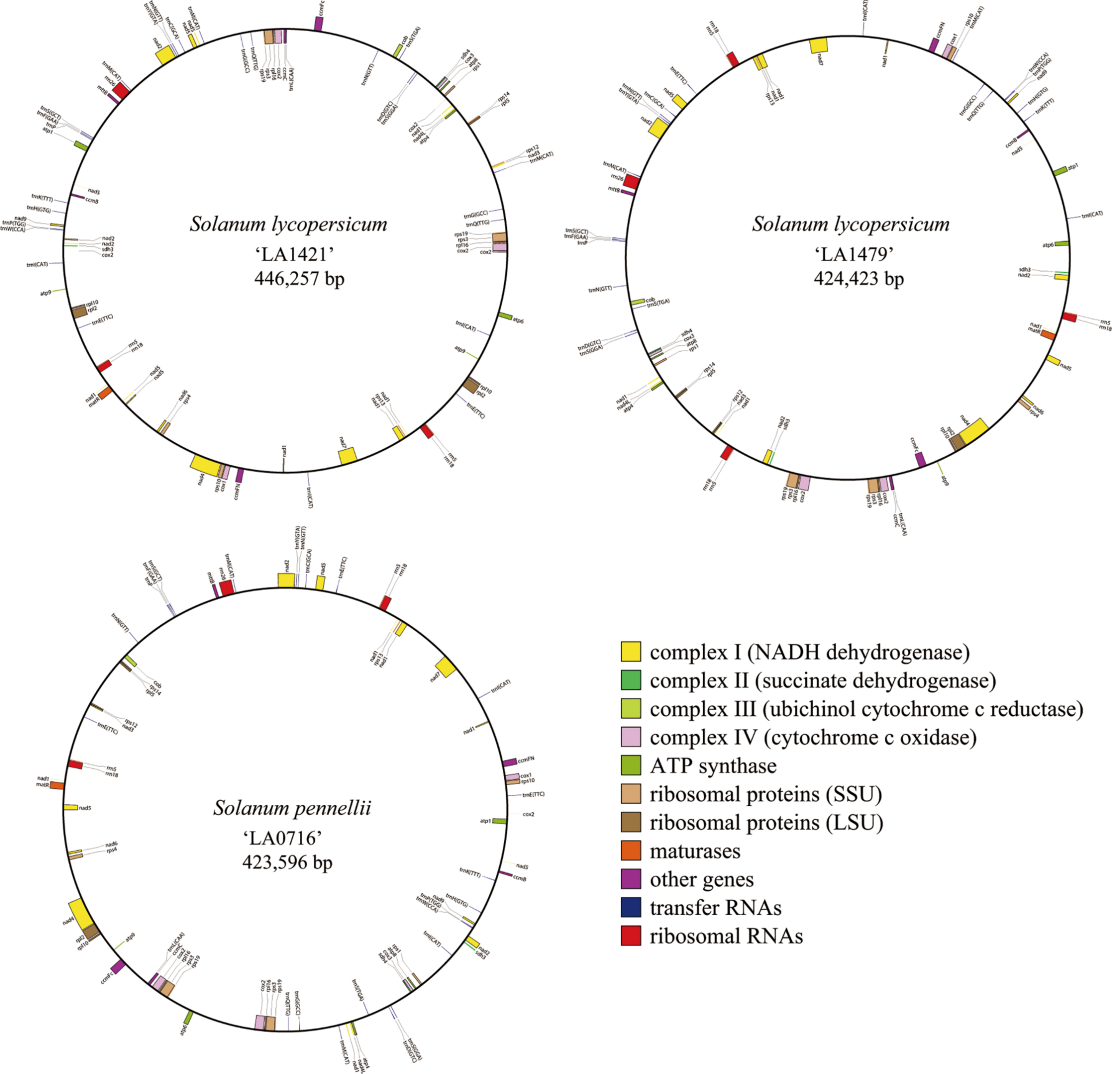
Maps of the three mitogenomes of the two tomato species.

**Fig 2:**
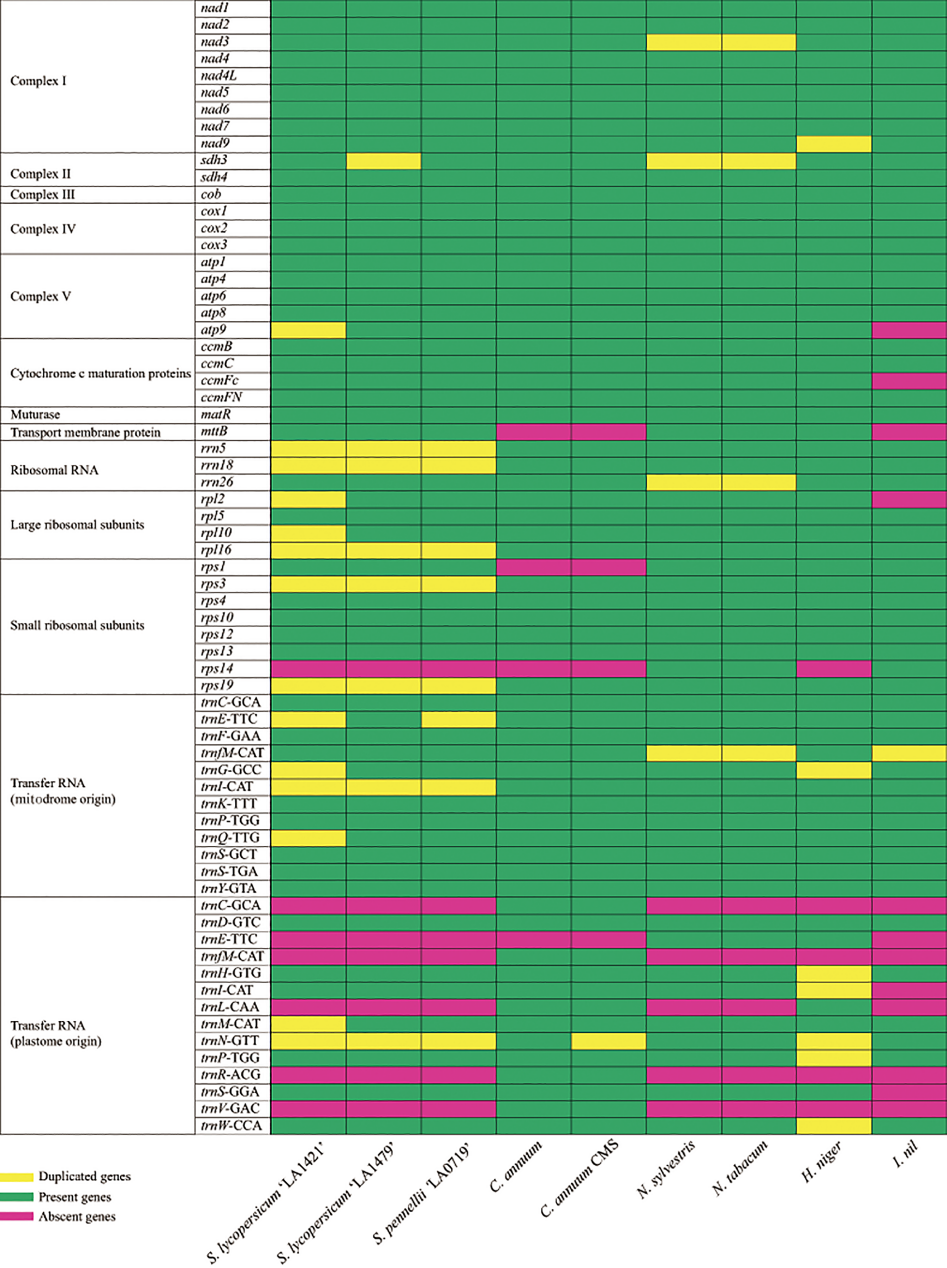
Genes present in the mitogenomes of tomato species and related species belonging to the Solanales.

The three mitogenome sequences were 423,596–446,257 bp in length (Fig 1 and Table 1). Their lengths were similar to the length of the MSA1 mitogenome which were generated by cell fusion between the tomato and potato [58] but were longer than that of the first draft of the tomato mitogenome [73]. Structurally, their GC content (45.0–45.2%) was similar to that of *Nicotiana sylvestris*, *N*. *tabacum* (45%), and *Capsicum annuum* (44.5%) (Table 1). The duplicated regions of the tomato mitogenome were 42,193–76,436 bp in length, and longer than those of other Solanales mitogenomes (Table 1). Thirty-seven coding genes, three rRNAs, and 20 tRNAs were identified in the three tomato mitogenomes (Fig 2). Among them, 5–8 genes were duplicated, excluding ORFs and tRNAs (Fig 2), whereas only 0–3 genes were duplicated in the mitogenomes of other plants belonging to the Solanales. Specifically, *rpl16*, *rps3*, *rps19*, *rrn5*, and *rrn18* were duplicated in the tomato mitogenomes, and *rps7* and *rps14* were deleted from the mitogenomes of tomato and other Solanaceae plants.

**Table 1:** Characteristics of the mitogenome sequences of tomato species and related species belonging to the Solanales.

Species	SRA Accession number	GenBank Accession number	GC content (%)	Total length (bp)	Length of duplicated regions (bp)
*Solanum lycopersicum* **‘LA1479’**	ERR418122^a^	MF034193	45.20	424,423	52,195
*Solanum lycopersicum* **‘LA1421’**	ERR418120 ^a^	MF034192	45.10	446,257	76,436
*Solanum* ***pennellii*** **‘LA0716’**	ERR418107 ^a^	MF034194	45.00	423,596	42,193
*Capsicum annuum* **‘Jeju’**		NC_024624	44.50	511,530	35,356
*Capsicum annuum* **CMS line FS4401**		KJ865409	44.50	507,452	21,770
*Nicotiana sylvestris*		NC_029805	45.00	430,597	35,890
*Nicotiana tabacum*		NC_006581	45.00	430,597	36,209
*Hyoscyamus niger*		NC_026515	45.10	501,401	41,500
*Ipomoea nil*		NC_031158	44.40	265,768	4,492

^a^These data were submitted by the 100 Tomato Genome Sequencing Consortium [59].

**Fig 3:**
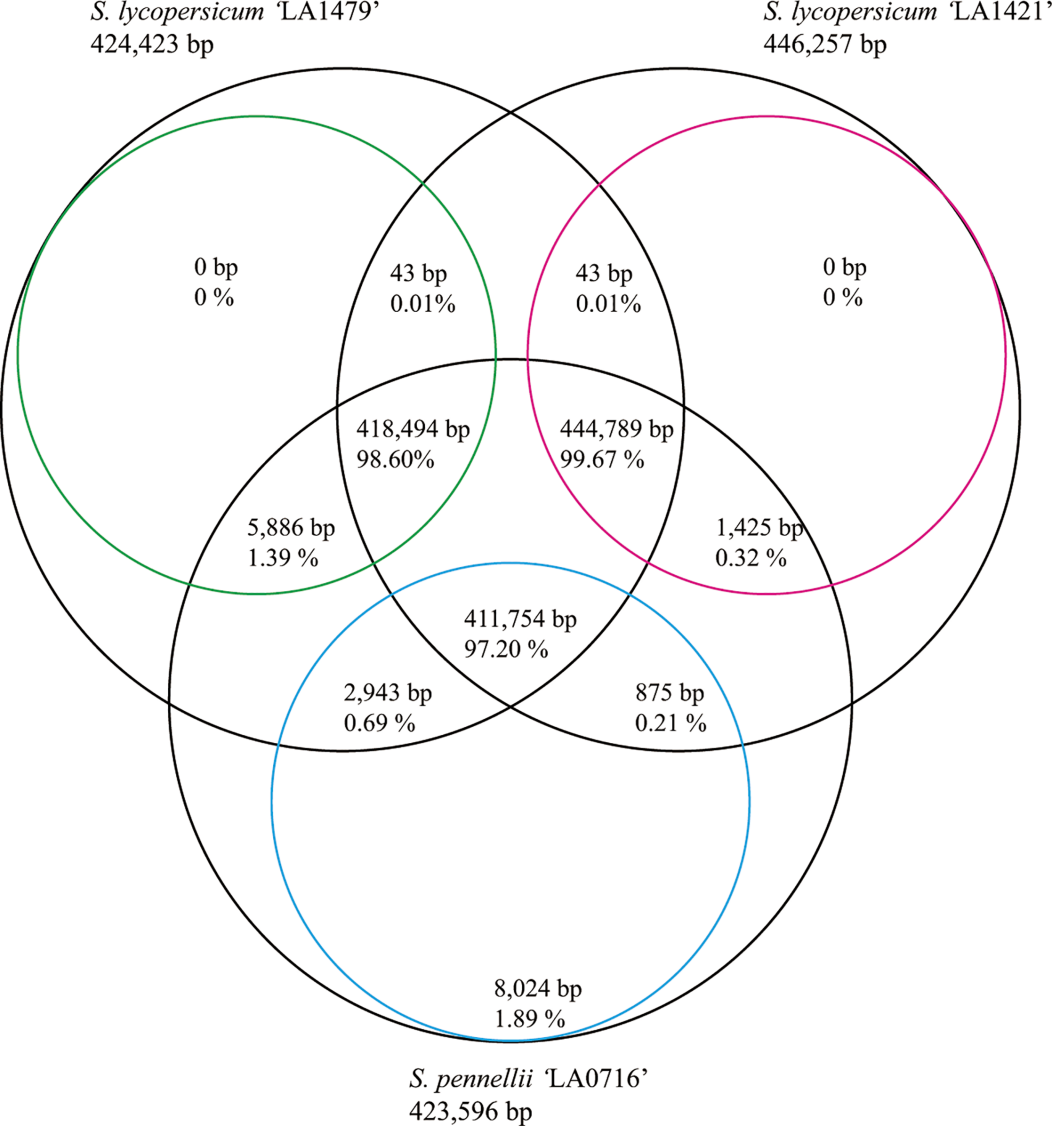
Conserved regions among the three tomato mitogenomes. Black circles represent the three tomato mitogenomes. Because of duplicated regions, conserved region lengths were not identical to that of each mitogenome. Colored circles indicate conserved region lengths for each mitogenome: green for *Solanum lycopersicum* ‘LA1479,’ red for *Solanum lycopersicum* ‘LA1421,’ and blue for *Solanum pennellii* ‘LA0716’.

**Fig 4:**
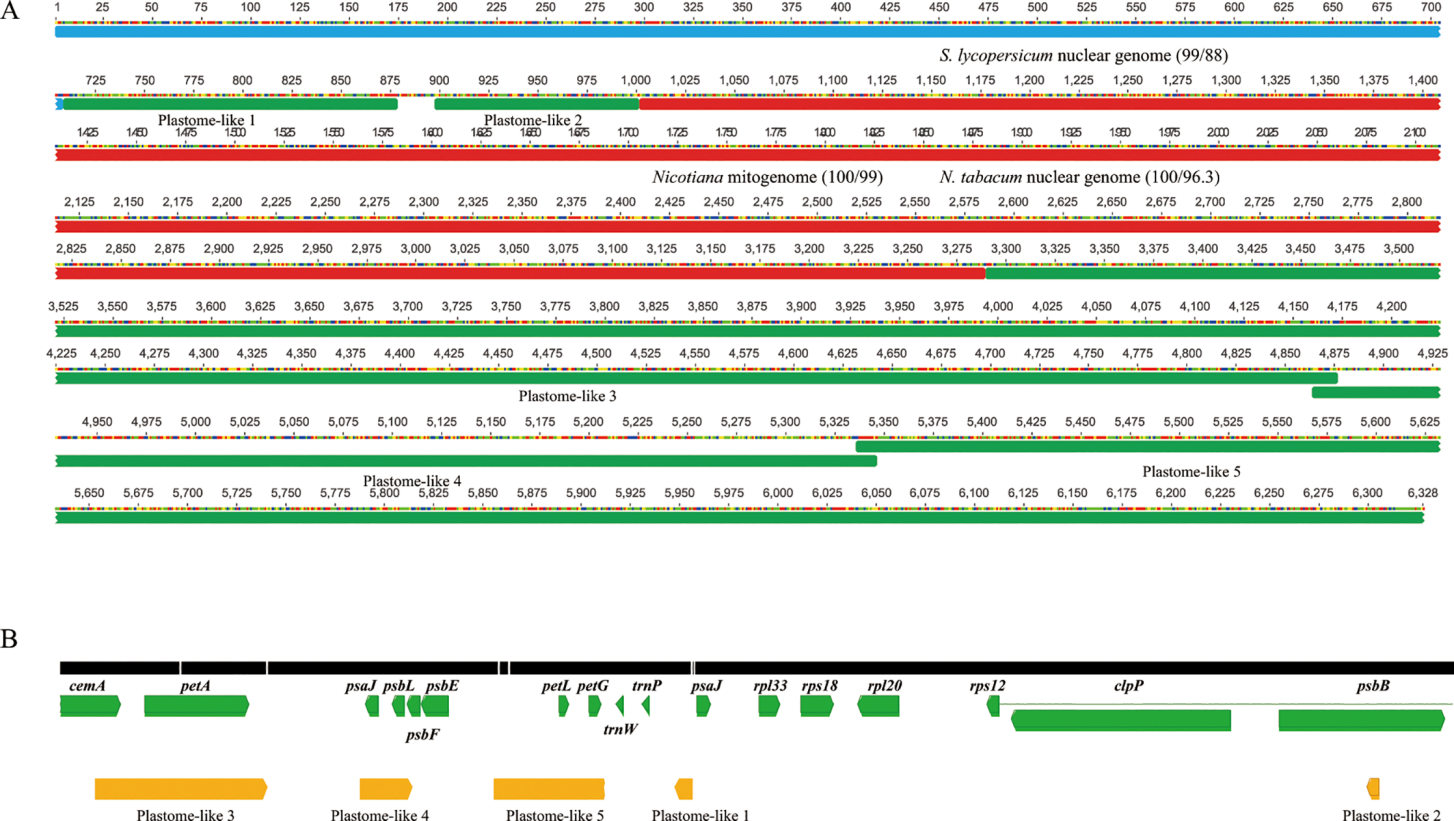
A 6,328-bp region specific to the *Solanum pennellii* mitogenome and plastome counterparts of five mitochondrial plastid DNAs (MTPTs). A) The 6,328-bp region comprised three parts: five MTPTs (green boxes), a region similar to the tomato nuclear genomic region (blue box), and regions similar to the mitogenome and nuclear genome of *Nicotiana tabacum* (red box). Numbers within parenthesis represent the query cover and identity, respectively, of a similar taxon using blastn analysis. B) Five MTPTs assembled in a plastome sequence (black bar). The green and yellow boxes indicate plastome genes and plastome-like regions, respectively.

Over 97% of the mitogenome sequences were conserved among the three tomato mitogenomes (Fig 3). The sequences of the two *S*. *lycopersicum* mitogenomes shared similarity with that of *S*. *pennellii* ‘LA0716,’ excluding a 43-bp region, whereas only four regions (223-, 460-, 1,013-, and 6,328-bp regions) were observed in the *S*. *pennellii* ‘LA0716’ mitogenome. Among them, the 6,328-bp region was almost identical to the nuclear genome sequence of *S*. *pennellii* ‘LA0716,’ with certain insertions and deletions. In addition to the identical part to the *S*. *pennellii* nuclear genome, the 6,328-bp region comprised three segments (Fig 4A). The first segment was almost identical to the *S*. *lycopersicum* nuclear genome, the second was identical to the mitogenome and nuclear genome of *Nicotiana*, and half of it was similar to the nuclear genomes of *S*. *lycopersicum* and *C*. *annuum*. The third segment comprised five plastome-like regions that corresponded with the tomato plastome (Fig 4B).

**Fig 5:**
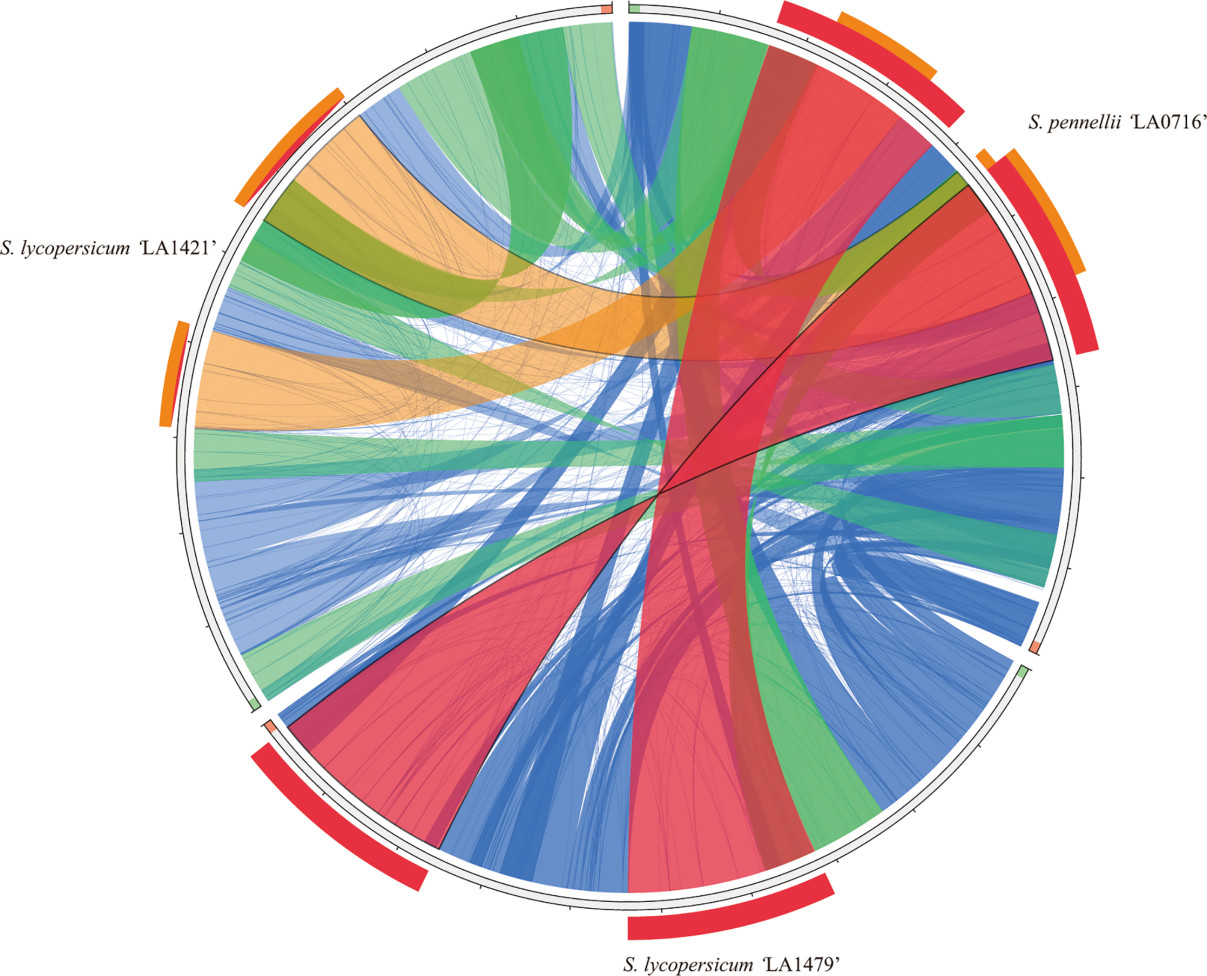
Map of rearrangements among the three tomato mitogenomes. Colors refer to the score/max bits core ratio, with blue ≤0.25, green ≤0.50, orange ≤0.75, and red >0.75.

There were numerous inter- and intraspecific mitogenome rearrangements; however, most of the mitogenome sequence regions in the three tomatoes were shared (Fig 5). Interestingly, the maximum syntenic region between *S*. *pennellii* ‘LA0716’ and *S*. *lycopersicum* ‘LA1479’ was higher than that between *S*. *lycopersicum* ‘LA1421’ and ‘LA1479’. Nineteen syntenic blocks were conserved among the three mitogenomes (S3 Table). The longest syntenic block was 61,213 bp in length and contained five genes, and the shortest was 5,815 bp in length and contained *sdh3* and exon1 and 2 of *nad2*.

### Duplicated regions in the tomato mitogenomes

The duplicated regions in the tomato mitogenomes ranged from 42,193 bp (*S*. *pennellii* ‘LA0716’) to 76,436 bp (*S*. *lycopersicum* ‘LA1421’) in length, and were longer than those of other Solanaceae species (Table 1). Compared with the other 56 core eudicot mitogenomes [74], the total duplicated regions of the three tomato mitogenomes were the 6^th^, 10^th^, and 12^th^ longest (S2 Fig). The correlation between the total duplicated region length and total mitogenome length was not significant according to the Pearson’s correlation coefficient (p = 0.2831). However, the total duplicated region length was significantly correlated with the maximum duplicated region length (p < 2.2e^−16^) with R^2^ = 0.72 (S2 Fig).

### Intracellular gene transfer from the plastome to the mitogenome

In total, 9,598 bp [large single copy of 2,558 bp, small single copy (SSC) of 32 bp, and an inverted repeat of 7,008 bp] of plastome sequences were detected in the three tomato mitogenomes, and a few of them were duplicated in each mitogenome. Mitochondrial plastid DNAs (MTPTs) in the tomato mitogenomes were 9,750–12,983 bp in length, constituting 2.2–3.1% of each mitogenome (Table 2). Compared with other Solanales species, the percentage of MTPTs in the tomato mitogenomes was more similar to that in *Nicotiana* and *Hyoscyamus* than in *Capsicum*, which is phylogenetically closer to *Solanum* [75]. However, most of the tomato MTPTs, excluding the partial sequences of *rps20*, *rps12*, and *ycf2*, were similar to the *C*. *annuum* MTPTs [34]. The SSC regions of the Solanaceae plastomes were highly conserved for transfer to mitogenomes, whereas the mitogenome of *Ipomoea nil*, belonging to the Solanales and the Solanaceae, contained a large SSC region.

**Table 2:** Regions transferred from the plastome to the mitogenome in the Solanales.

	Regions transferred from plastome to mitogenome			Total length of regions transferred from plastome to mitogenome (bp)	Percentage of plastome regions in mitogenome (%)
Species	LSC (bp)	SSC (bp)	IR (bp)		
*Solanum lycopersicum* **‘LA1421’**	2,558	32	7,008	9,774	2.2
*Solanum lycopersicum* **‘LA1479’**	2,558	32	7,008	9,750	2.3
*Solanum pennellii* **‘LA0716’**	5,936	32	7,008	12,983	3.1
*Capsicum annum* **CMS**	33,212	32	18,767	53,480	10.5
*Capsicum* ***annum* non-CMS**	33,398	32	21,238	57,215	11.2
*Hyoscyamus niger*	6,334	79	4,536	12,627	2.5
*Nicotiana sylvestris*	4,396	0	6,036	10,773	2.5
*Nicotiana tabacum*	4,396	0	6,306	10,772	2.5
*Ipomoea nil*	16,397	3,602	7,866	28,479	10.7

LSC, large single copy; SSC, small single copy; IR, inverted repeat.

As mentioned above, only five MTPTs were observed in the *S*. *pennellii* ‘LA0716’ mitogenome among the three tomato mitogenomes, and the plastome counterparts of the five MTPTs were located nearby, excluding the partial *psbB* region (Fig 4B). To further analyze these five MTPTs, they were grouped into sequence A, which included two small plastome regions, and sequence B, which included three large plastome regions (S3 Fig). Sequence A was observed in the *C*. *annuum* mitogenome, whereas sequence B was only partly observed in certain angiosperm mitogenomes (S3 Fig). Interestingly, the mitogenome of *Hesperelaea palmeri* [76], which is an extinct Oleaceae species, shared four MTPTs with that of *S*. *pennellii* ‘LA0716’, although the similarity in MTPTs between the *S*. *pennellii* ‘LA0716’ and *H*. *palmeri* mitogenomes was weaker than that between the *S*. *pennellii* ‘LA0716’ and *Nicotiana* mitogenomes.

By comparing the nuclear genomes of *Solanum* and *Capsicum* using blastn [65], numerous sequence-A-similar regions were observed in the nuclear genomes of *Solanum* and *C*. *annuum*, excluding that of *S*. *lycopersicum* ‘Heinz1706,’ which had only one region (S4 Fig). Although the phylogenetic tree constructed using Bayesian inference did not completely determine the relationship among sequence-A-similar regions in the *Solanum* and *C*. *annuum* genomes (data not shown), sequence-A-similar regions in the *C*. *annuum* genome were distinguished from those in the *Solanum* genomes by two deletions (4 bp and 2 bp) (S4 Fig). Sequence-B-similar regions were not observed in the nuclear genomes of *S*. *lycopersicum*, *S*. *tuberosum*, or *C*. *annuum*.

### Gene transfer between the mitogenome and nuclear genome

Most of the sequences shared between the tomato mitogenome and nuclear genome were not coding regions, and noncoding regions in mitogenomes vary among land plant species. Therefore, it was difficult to determine the direction of gene transfer between the mitogenome and nuclear genome. Consequently, all of the sequences shared between the tomato mitogenome and nuclear genome were considered NUMTs.

In total, 15,670–16,844 NUMTs were observed in the nuclear genomes of *S*. *pennellii* ‘LA0716’ and *S*. *lycopersicum* ‘Heinz1706’ (Table 3). The total length of NUMTs in the *S*. *pennellii* ‘LA0716’ nuclear genome (3,412 kb) was greater than that in the *S*. *lycopersicum* ‘Heinz1706’ (2,944 kb) nuclear genome, representing 0.37% of the total nuclear genome. Most of the NUMTs in the two tomato species were observed on chromosome 1; however, they occupied less than 0.29–0.32% of it. In contrast, 0.72% and 0.74% of chromosome 11 in the two species was homologous to their mitogenomes.

**Table 3:** Nuclear copies of mitochondrial DNA (NUMTs) in the nuclear genomes of tomato (*Solanum*) species.

Comparison	Position	No. of NUMTs	Mean length (bp)	Median length (bp)	Maximum length (bp)	Minimum length (bp)
mitogenom**e of *S*. *pennellii ‘*LA0716’** ***vs*** **nuclear genome of *S*. *pennellii ‘*LA0716’**	ch01	2,015 (233)^a^	175.4 (711.6)	102 (397)	13,387	36 (250)
	ch02	1,211 (160)	193.5 (752.5)	108 (356)	8,035	36 (250)
	ch03	1,245 (107)	137.5 (440.5)	103 (327)	2,985	36 (250)
	ch04	1,886 (110)	131.8 (515.4)	96 (328.5)	3,531	36 (250)
	ch05	1,327 (181)	292.5 (1,452.8)	108 (388)	18,135	37 (251)
	ch06	1,107 (115)	204.2 (1,012)	106 (354)	14,969	36 (253)
	ch07	1,298 (132)	163.5 (648.1)	105 (355.5)	6,483	37 (250)
	ch08	1,199 (126)	203.3 (1,033.1)	98 (352.5)	15,852	36 (250)
	ch09	1,317 (145)	183.2 (821.2)	98 (365)	8,261	36 (250)
	ch10	1,366 (194)	231.6 (964.8)	107 (430)	10,180	36 (253)
	ch11	1,487 (245)	330.6 (1,457.7)	111 (405)	22,940	36 (253)
	ch12	1,386 (137)	205.8 (1,097)	103.5 (389)	23,276	36 (250)
mitogenome **of *S*. *lycopersicum ‘*LA1479’** ***vs*** **nuclear genome of *S*. *lycopersicum* ‘Heinz1706’**	ch01	1,694 (206)	169.7 (610.7)	105 (379.5)	6,628	36 (250)
	ch02	1,099 (121)	165.2 (621.4)	107 (346)	4,128	36 (251)
	ch03	1,417 (191)	198.7 (777.8)	107 (355)	7,242	36 (251)
	ch04	1,452 (88)	130.7 (489.2)	96 (329.5)	3,226	36 (250)
	ch05	1,330 (178)	216.1 (910.2)	105.5 (361)	12,271	36 (250)
	ch06	1,090 (98)	132.3 (395.8)	100.5 (327)	1,720	36 (250)
	ch07	1,203 (128)	162.8 (628.8)	104 (367)	5,091	36 (250)
	ch08	1,157 (124)	177.2 (748.1)	104 (321.5)	8,393	36 (250)
	ch09	1,341 (108)	177.3 (1,026.1)	97 (349)	11,571	36 (252)
	ch10	1,243 (144)	212.2 (983.1)	107 (417)	12,227	37 (251)
	ch11	1,395 (224)	291.8 (1,238.3)	111 (447)	29,977	36 (251)
	ch12	1,296 (156)	202.6 (885.4)	106 (363.5)	19,816	36 (250)
mitogenome **of *S*. *lycopersicum ‘*LA1421’** ***vs*** **nuclear genome of *S*. *lycopersicum* ‘Heinz1706’**	ch01	1,688 (207)	169.9 (610.3)	105 (378)	6,950	36 (250)
	ch02	1,095 (119)	165.6 (631.8)	107 (341)	7,167	36 (251)
	ch03	1,417 (190)	199.2 (783.9)	107 (355)	7,340	36 (251)
	ch04	1,449 (89)	130.9 (488.4)	96 (329)	3,226	36 (250)
	ch05	1,324 (175)	218.3 (937.2)	105 (360)	12,271	36 (250)
	ch06	1,085 (96)	132 (397)	101 (327)	1,720	36 (250)
	ch07	1,204 (126)	163 (641)	104 (363)	5,091	36 (250)
	ch08	1,147 (120)	178.3 (771.1)	104 (321.5)	8,884	36 (250)
	ch09	1,340 (108)	177.4 (1,026.1)	98 (349)	11,571	36 (252)
	ch10	1,247 (146)	212 (974.4)	107 (414)	12,227	37 (251)
	ch11	1,382 (217)	277.3 (1,167.9)	111 (442)	31,315	36 (251)
	ch12	1,292 (154)	203.9 (904)	106.5 (358.5)	19,816	36 (250)

^a^Numbers within parenthesis represent the respective values for NUMTs longer than 250 bp.

**Fig 6:**
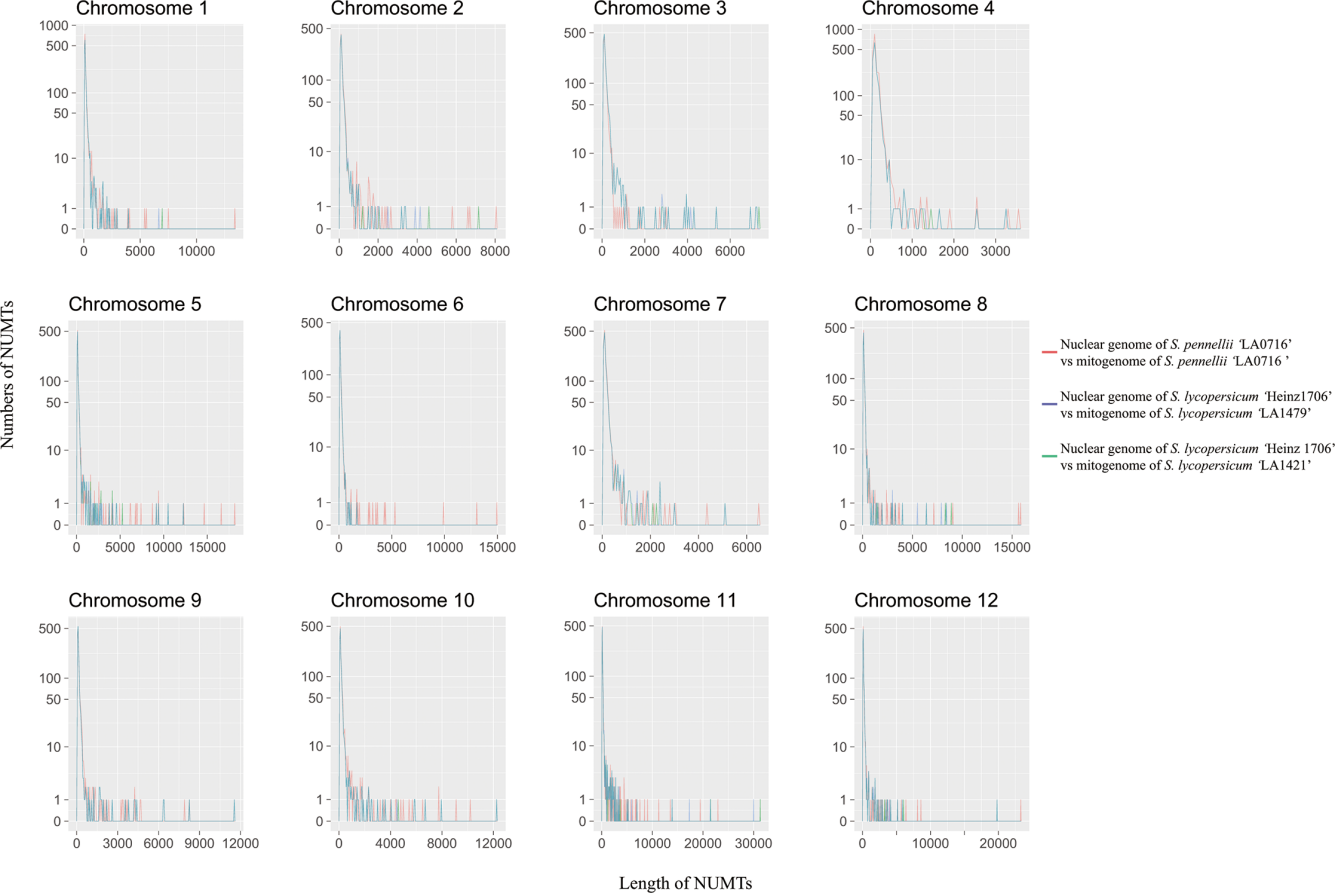
Relationship between the length and number of nuclear copies of mitochondrial DNA (NUMTs) in 12 tomato chromosomes.

NUMTs were evenly distributed among the chromosomes (S5–S7 Figs). However, NUMTs longer than 1,000 bp were tightly clustered, and regions containing numerous large NUMTs were not identical between the two nuclear genomes of *S*. *pennellii* ‘LA0716’ and *S*. *lycopersicum* ‘Heinz1706’. Specifically, the number of NUMTs longer than 5,000 bp in *S*. *pennellii* ‘LA0716’ was nearly twice that in *S*. *lycopersicum* ‘Heinz1706’ (Fig 6). Half of the large NUMTs in *S*. *pennellii* ‘LA0716’ were on chromosomes 5 and 11. Consequently, the median length of the NUMTs on chromosomes 5 and 11 in *S*. *pennellii* ‘LA0716’ was similar to that in *S*. *lycopersicum*; however, the mean length of the NUMTs on chromosomes 5 and 11 in *S*. *pennellii* ‘LA0716’ was greater than that in *S*. *lycopersicum* ‘Heinz1706’ (Table 3).

### Gene transfer from the plastome to the nuclear genome

There were 7,445 and 7,805 NUPTs in the nuclear genomes of *S*. *lycopersicum* ‘Heinz1706’ and *S*. *pennellii* ‘LA0716,’ respectively (S4 Table). The cumulative NUPT length was 1,533,904–1,739,535 bp, constituting 0.189–0.191% of the nuclear genome. The cumulative NUPT lengths of chromosome 1 in *S*. *lycopersicum* ‘Heinz1706’ and chromosome 10 in *S*. *pennellii* ‘LA0716’ were the longest among the chromosomes, and occupied 0.28% and 0.40% of each chromosome in *S*. *lycopersicum* and *S*. *pennellii*, respectively. Similar to the NUMTs, large NUPTs were tightly clustered; however, their locations were not identical in the two tomato nuclear genomes (S8 and S9 Figs).

### Similarity and structural mutations in nuclear-transferred organellar DNA and counterparts

To determine why large organellar copies were clustered in certain nuclear genome loci, two assumptions were made. The first was that NUPTs and NUMTs that were tightly clustered originated from the DNA of a single organelle, and the second was that the occurrence of structural mutations, such as rearrangements and insertions/deletions, and base substitutions had increased with time after EGT from organellar genomes to the nuclear genome.

Large NUPTs (longer than 1 kb) were tightly clustered in 11 regions of the *S*. *pennellii* ‘LA0716’ nuclear genome (S10 Fig). In these 11 regions, certain NUPTs that appeared to be more structurally mutated because of large inversions, rearrangements, or insertions/deletions had less similarity with their counterparts than those that appeared to be less structurally mutated because of small insertions/deletions or duplications. However, certain NUPTs that appeared to be more structurally mutated had stronger similarity than those that appeared to be less structurally mutated.

Large, tightly clustered NUMTs (longer than 1 kb) were observed in 24 regions of the *S*. *pennellii* ‘LA0716’ nuclear genome (S11 Fig). Similar to the large NUPTs, certain NUMTs that appeared to be more structurally mutated had a lower similarity to their counterparts than those that appeared to be less structurally mutated. However, certain regions with numerous large insertions/deletions or inversions had over 99.4% similarity compared with their counterparts. In particular, the similarities of large NUMTs on chromosome 5 in *S*. *pennellii* ‘LA0716’ were over 96.1%, although large deletions and rearrangements appeared to have occurred.

## Discussion

### Structural variations in tomato mitogenomes

Considering the slow evolutionary rates of sequence [11] and gene [77] conservation in plant mitogenomes, the low similarity between plant mitogenomes of closely related genera [9, 78, 79] and foreign DNA causing variation in plant mitogenome length [80–82] suggest that the mitogenomes of land plants appear to comprise syntenic blocks containing coding genes and unique regions that contain noncoding regions, and these unique regions appear to be related to foreign DNA. In addition, 59 eudicot mitogenome sequences, including the three tomato mitogenomes, showed that there was no correlation between total duplicated region length and mitogenome sequence length (S2 Fig). Therefore, it appears that foreign DNA has more effect than duplicated regions on length variations at high taxonomic levels (family and above).

However, duplication appears to be a cause of mitogenome expansion at low taxonomic levels, such as inter- and intraspecific levels. Except for the duplicated regions, 97.1–99.3% of the mitogenome sequences of *Brassica juncea* (219,766 bp) and *Brassica oleracea* (360,271 bp) could be aligned together [14]. The lengths of the tomato mitogenomes were strongly related to the duplicated regions. The duplicated regions in the *S*. *lycopersicum* ‘LA1421’ mitogenome were the largest among the three tomato mitogenomes (Table 1). The total length of the duplicated regions in the *S*. *lycopersicum* ‘LA1479’ mitogenome was 23% greater than that in the *S*. *pennellii* ‘LA0716’ mitogenome. Consequently, the two *S*. *lycopersicum* mitogenomes were larger than that of *S*. *pennellii* ‘LA0716,’ although the *S*. *pennellii* ‘LA0716’ mitogenome contained a unique 8,024-bp sequence compared with the two *S*. *lycopersicum* mitogenomes. Because the maximum duplicated region length was significantly correlated with the total duplicated region length (S2 Fig), the total length difference between mitogenomes of closely related taxa seem to be more affected by the maximum duplicated region than by short duplicated regions. In contrast to plastomes, the structures of mitogenomes in land plants have evolved rapidly [11], and direct repeats and inverted repeats have facilitated rearrangements [77, 83]. Therefore, the mitogenomes of land plants probably evolved to be able to produce duplications frequently and easily in order to rapidly alter their structures.

Rearrangement is a major issue in studies on mitogenomes, because it can result in the generation of novel chimeric ORFs, which is a new, related phenomenon [78, 84]. Many previous studies have demonstrated great intraspecific [78, 85–87] and interspecific [10, 14] variability in mitogenome structure. Numerous repeat regions support the possibility of a greater number of rearrangements in tomato mitogenomes, because recombination via inverted repeats and direct repeats induces the inversion of intervening sequences and subgenomic molecules, respectively [17, 83, 88].

### Gene transfer from the plastome to the mitogenome via the nuclear genome

Among the five MTPTs detected only in the *S*. *pennellii* ‘LA0716’ mitogenome among the three tomato strains, the entire sequence A region was detected in the *C*. *annuum* mitogenome (S3 Fig); however, the plastome counterparts were distant (Fig 4B). According to Wang et al. [34], in 39 seed plants, MTPT gene clusters containing *psbB* did not contain *psaJ*. Therefore, it is probable that two small plastome regions were integrated together in the ancestor of *Solanum* and *Capsicum*. Subsequently, the sequence A region might have transferred into the mitogenomes of both *S*. *pennellii* and *C*. *annuum*. If this scenario is correct, why was this region only transferred to the *S*. *pennellii* and *C*. *annuum* mitogenomes and not to the *S*. *lycopersicum* mitogenome? Because the sequence A region was observed in the nuclear genomes of *Solanum* species and *C*. *annuum* (S4 Fig), this region might have initially infiltrated the nuclear genome of the common ancestor of *Solanum* and *Capsicum*. Subsequently, the sequence A region was duplicated in the nuclear genomes of both *S*. *pennellii* and *C*. *annuum*, but not in *S*. *lycopersicum*, after the speciation of extant tomato species. According to a recent phylogenetic study on the Solanaceae [89], the ancestor of *Capsicum* diverged from that of *Solanum* 19.13 Ma, and the ancestor of *S*. *pennellii* diverged from that of *S*. *lycopersicum* 1.72 Ma. Therefore, it appears that the first infiltration of integrated plastome sequences dates back at least to the Neogene, and sequence A region duplications occurred during the Quaternary period. Because there were frequent gene transfers between the mitogenome and nuclear genome during evolution [23, 26, 90, 91], the presence of multiple copies of sequence A in the nuclear genomes of *S*. *pennellii* and *C*. *annuum* could have increased their chances of transfer into the mitogenome, compared with one sequence A copy in the *S*. *lycopersicum* genome. Therefore, the sequence A region in the mitogenomes of *S*. *pennellii* and *C*. *annuum* appears to have been independently transferred from the nuclear genome, and this finding indicates that certain MTPTs are the result of two-step gene transfers, i.e., plastome → nuclear genome → mitogenome.

### Recent EGTs from organellar genomes to the nuclear genome *vs* rapid deletion of organellar copies from the nuclear genome

Original, large insertions appear to have been degraded during evolution into smaller fragments [22]. Therefore, the total length of organellar DNA copies in the nuclear genome was negatively correlated with the number of organellar DNA fragments, because of degradation during evolution.

The total numbers of NUPTs and NUMTs in *S*. *pennellii* were similar to those in *S*. *lycopersicum;* however, their cumulative lengths were 13% and 16% longer, respectively, in *S*. *pennellii*. These variations in cumulative length were caused by large organellar copies in the nuclear genome of *S*. *pennellii*. In addition, a discordance of NUPTs between two *Oryza* subspecies has also been reported [46].

The lengths and numbers of large organellar DNA fragments in the *S*. *lycopersicum* nuclear genome were lower than those in the *S*. *pennellii* nuclear genome. If the evolutionary rate of the decrease and deletion of insertion fragments in *S*. *lycopersicum* was greater than that in *S*. *pennellii*, it is clear why there were more, and larger, organellar copies in *S*. *pennellii* than in *S*. *lycopersicum*.

However, if the difference in the number of large fragments was caused entirely by the different evolutionary rates of the decrease and deletion of insertion fragments between the two tomatoes, a similar ratio of large fragments in each chromosome of *S*. *pennellii* and *S*. *lycopersicum* might be achieved. However, the distribution of large NUMTs (longer than 3,000 bp) in chromosome 3 differed to that in chromosome 6 in the two tomatoes. Therefore, the assumption that recent EGTs occurred between organellar DNA and the nuclear genome can be accepted.

Consequently, it appears that the evolutionary rates of the decrease and deletion of fragments inserted in the nuclear genome were altered in the two tomatoes after they diverged, and recent EGTs differentiated the distribution of organellar copies on certain chromosomes to that on other chromosomes.

### Clustered, large organellar DNA in the nuclear genome

Long NUPT and NUMT fragments were frequently found in different regions of the nuclear genomes of the same tomato species. Few of these large organellar DNA fragments appeared to have originated from long organellar genomic fragments, because they were derived from closely located regions of the organellar genomes, and showed high similarity with their organellar genome counterparts. Michalovova et al. [90] suggested that new organellar DNA sequences were inserted near centromeres, degraded by transposable elements, and then scattered by structural mutations. However, certain nuclear regions with long organellar DNA fragments were derived from different regions of the plastome or mitogenome, and did not appear to be older than the long fragments derived from organellar DNA in terms of sequence divergence (S10 and S11 Figs). These mosaic organellar DNA fragments cannot be explained by the single organellar DNA origin hypothesis, which is based on the similarity between organelle-derived nuclear DNA and organellar DNA without structural mutations [22].

Noutsos et al. [24] suggested that mosaic organellar DNA fragments were generated by 1) the random end-joining of different fragments before integration; 2) rapid rearrangements after integration; or 3) by the ongoing integration of organellar DNA at the same locus. The first and second scenarios explain the clustered, large NUPTs and NUMTs in tomato nuclear genomes; however, why were the loci of the clustered NUPTs and NUMTs distantly located? If the random end-joining of different fragments before integration occurred regardless of genomic source (mitogenome or plastome), large fragments of the mitogenome and plastome could have merged, like five complex insertions of *O*. *sativa* containing rearranged DNA from the mitogenome and plastome [24] and not like the tomato nuclear genome. The discordance between nuclear genomic regions with long plastome fragments and those with long mitogenome fragments could have been caused by very large, continuous integrants and consecutive rearrangements [24]; however, this hypothesis cannot apply to all nuclear organellar DNA copies, because certain organellar DNA copies that appear to be more structurally mutated had a stronger similarity with their counterparts than those that appear to be less structurally mutated. We could not identify hotspots of organellar DNA integration into the nuclear genome with the data available; however, the hotspot hypothesis could explain the discordance between nuclear genomic regions with long plastome fragments and those with long mitogenome fragments.

## Supporting information

S1 Fig Coverage depths of the mitogenomes and plastomes used in this study. Raw reads were mapped to mitogenomes and plastomes using Geneious aligner with zero mismatch and gap among the reads, and the Burrows-Wheeler alignment tool with the default options set to verify the coverage depths through the genome. Sharp peaks that were up to 20-fold higher than base coverage indicate mitochondrial plastome regions. Coverages were higher than 200, except for certain regions containing homopolymers or AT-rich regions, which had low coverage depth. However, these regions were also supported by numerous paired-end reads (blue bar and red line indicate paired-end reads and intervals between paired-end reads, respectively, in *Solanum lycopersicum* ‘LA1479’). The X-axis and Y-axis indicate positions and coverage depths, respectively.
(TIF)

S2 Fig Duplicated regions in 59 core eudicot mitogenomes. (A) Total mitogenome length *vs* total duplicated region length. (B) Maximum lengths of duplicated regions *vs* total lengths of duplicated regions. Green triangles represent the three tomato mitogenomes.
(TIF)

S3 Fig Mitogenomes aligned to a 6,328-bp region in *Solanum pennellii*. Species are divided by dashed red lines. The yellow box on top represents mitochondrial plastid DNAs. The gray regions on the other angiosperm chromosomes are more similar to the *S*. *pennellii* ‘LA0716’ mitogenome than the black regions.
(TIF)

S4 Fig Alignment of sequence-A-similar regions in *Solanum* and *Capsicum*. Numerous sequence-A-similar regions were observed in the *S*. *pennellii*, *S*. *tuberosum*, and *C*. *annuum* nuclear genomes; however, one sequence A copy was also observed in the *S*. *lycopersicum* ‘Heinz1706’ nuclear genome.
(TIF)

S5 Fig Nuclear copies of mitochondrial DNA (NUMTs) in *Solanum pennellii* ‘LA0716’. The X-axis indicates the positions of the NUMTs and the Y-axis indicates the lengths of the NUMTs on each chromosome of *S*. *pennellii* ‘LA0716’.
(TIF)

S6 Fig *Solanum lycopersicum* ‘LA1479’ mitogenome-like nuclear copies of mitochondrial DNA (NUMTs) in *S*. *lycopersicum* ‘Heinz1706’. The X-axis indicates the positions of the NUMTs and the Y-axis indicates the lengths of the NUMTs on each chromosome of *S*. *lycopersicum* ‘Heinz1706’.
(TIF)

S7 Fig *Solanum lycopersicum* ‘LA1421’ mitogenome-like nuclear copies of mitochondrial DNA (NUMTs) in *S*. *lycopersicum* ‘Heinz1706’. The X-axis indicates the positions of the NUMTs and the Y-axis indicates the lengths of the NUMTs on each chromosome of *S*. *lycopersicum* ‘Heinz1706’.
(TIF)

S8 Fig Nuclear copies of plastid DNA (NUPTs) in *Solanum pennellii* ‘LA0716’. The X-axis indicates the positions of the NUPTs and the Y-axis indicates the lengths of the NUPTs on each chromosome of *S*. *pennellii* ‘LA0716’.
(TIF)

S9 Fig *Solanum lycopersicum* ‘LA1479’ plastome-like nuclear copies of plastid DNA (NUPTs) in *S*. *lycopersicum* ‘Heinz1706’. The X-axis indicates the positions of the NUPTs and the Y-axis indicates the lengths of the NUPTs on each chromosome of *S*. *lycopersicum* ‘Heinz1706’.
(TIF)

S10 Fig Dot matrix analysis of the plastome (X-axis) and 11 nuclear regions including long nuclear copies of plastid DNA (NUPTs) fragments (Y-axis) in *Solanum pennellii*. The percentages located on the right-hand side of the boxes indicate the similarity between NUPTs (≥1,000 bp) and their counterparts in the plastome. The colored line at the bottom indicates the positions of large single copy (LSC), inverted repeat (IR), and small single copy (SSC) regions.
(TIF)

S11 Fig Dot matrix analysis of the mitogenome (X-axis) and 24 nuclear regions including long nuclear copies of mitochondrial DNA (NUMTs) fragments (Y-axis) in *Solanum pennellii*. The percentages located on the right-hand side of the boxes indicate the similarity between NUMTs (≥1,000 bp) and their counterparts in the mitogenome. The colored arrow below the bottom line indicates large repeat sequences (≥5,000 bp) in the mitogenome.
(TIF)

S1 Table Mitogenome sequences in core eudicots. (DOCX)

S2 Table Regions with less than 200 coverage depths in the three mitogenomes. (DOCX)

S3 Table Syntenic blocks in the three tomato mitogenomes. (DOCX)

S4 Table Nuclear copies of plastid DNA (NUPTs) in the nuclear genomes of tomato species. (DOCX)

## References

[1] Bock R, Knoop V. Genomics of chloroplasts and mitochondria: Springer Science & Business Media; 2012.

[2] Villarreal JC, Forrest LL, Wickett N, Goffinet B. The plastid genome of the hornwort Nothoceros aenigmaticus (Dendrocerotaceae): phylogenetic signal in inverted repeat expansion, pseudogenization, and intron gain. Am J Bot. 2013;100(3):467–77. Epub 2013/02/19. 10.3732/ajb.1200429 . 23416362

[3] Kim HT, Chung MG, Kim KJ. Chloroplast genome evolution in early diverged leptosporangiate ferns. Mol Cells. 2014;37(5):372–82. Epub 2014/05/16. doi: 10.14348/molcells.2014.2296 ; PubMed Central PMCID: PMCPMC4044308. 24823358 10.14348/molcells.2014.2296 PMC4044308

[4] Ruhlman TA, Jansen RK. The plastid genomes of flowering plants. Methods Mol Biol. 2014;1132:3–38. Epub 2014/03/07. 10.1007/978-1-62703-995-6_1 . 24599844

[5] Kim JS, Kim HT, Kim J-H. The Largest Plastid Genome of Monocots: a Novel Genome Type Containing AT Residue Repeats in the Slipper Orchid Cypripedium japonicum. Plant Mol Biol Rep. 2014;33(5):1210–20. 10.1007/s11105-014-0833-y

[6] Delannoy E, Fujii S, Colas des Francs-Small C, Brundrett M, Small I. Rampant gene loss in the underground orchid Rhizanthella gardneri highlights evolutionary constraints on plastid genomes. Mol Biol Evol. 2011;28(7):2077–86. Epub 2011/02/04. 10.1093/molbev/msr028 ; PubMed Central PMCID: PMCPMC3112369. 21289370 PMC3112369

[7] Chumley TW, Palmer JD, Mower JP, Fourcade HM, Calie PJ, Boore JL, et al The complete chloroplast genome sequence of Pelargonium x hortorum: organization and evolution of the largest and most highly rearranged chloroplast genome of land plants. Mol Biol Evol. 2006;23(11):2175–90. Epub 2006/08/19. 10.1093/molbev/msl089 . 16916942

[8] Liu Y, Medina R, Goffinet B. 350 my of mitochondrial genome stasis in mosses, an early land plant lineage. Mol Biol Evol. 2014;31(10):2586–91. Epub 2014/07/02. 10.1093/molbev/msu199 . 24980738

[9] Naito K, Kaga A, Tomooka N, Kawase M. De novo assembly of the complete organelle genome sequences of azuki bean (Vigna angularis) using next-generation sequencers. Breed Sci. 2013;63(2):176–82. Epub 2013/07/16. 10.1270/jsbbs.63.176 ; PubMed Central PMCID: PMCPMC3688379. 23853512 PMC3688379

[10] Tang M, Chen Z, Grover CE, Wang Y, Li S, Liu G, et al Rapid evolutionary divergence of Gossypium barbadense and G. hirsutum mitochondrial genomes. BMC Genomics. 2015;16(1):770 Epub 2015/10/16. 10.1186/s12864-015-1988-0 ; PubMed Central PMCID: PMCPMC4603758. 26459858 PMC4603758

[11] Palmer JD, Herbon LA. Plant mitochondrial DNA evolved rapidly in structure, but slowly in sequence. J Mol Evol. 1988;28(1–2):87–97. 3148746 10.1007/BF02143500

[12] Adams KL, Qiu YL, Stoutemyer M, Palmer JD. Punctuated evolution of mitochondrial gene content: high and variable rates of mitochondrial gene loss and transfer to the nucleus during angiosperm evolution. Proc Natl Acad Sci U S A. 2002;99(15):9905–12. Epub 2002/07/18. 10.1073/pnas.042694899 ; PubMed Central PMCID: PMCPMC126597. 12119382 PMC126597

[13] Adams KL, Daley DO, Whelan J, Palmer JD. Genes for two mitochondrial ribosomal proteins in flowering plants are derived from their chloroplast or cytosolic counterparts. Plant Cell. 2002;14(4):931–43. 10.1105/tpc.010483 WOS:000175350100016. 11971146 PMC150693

[14] Chang S, Yang T, Du T, Huang Y, Chen J, Yan J, et al Mitochondrial genome sequencing helps show the evolutionary mechanism of mitochondrial genome formation in Brassica. BMC Genomics. 2011;12(1):497 Epub 2011/10/13. 10.1186/1471-2164-12-497 ; PubMed Central PMCID: PMCPMC3204307. 21988783 PMC3204307

[15] Andre C, Levy A, Walbot V. Small repeated sequences and the structure of plant mitochondrial genomes. Trends Genet. 1992;8(4):128–32. Epub 1992/04/01. 10.1016/0168-9525(92)90370-J . 1631955

[16] Alverson AJ, Wei X, Rice DW, Stern DB, Barry K, Palmer JD. Insights into the evolution of mitochondrial genome size from complete sequences of Citrullus lanatus and Cucurbita pepo (Cucurbitaceae). Mol Biol Evol. 2010;27(6):1436–48. Epub 2010/02/02. 10.1093/molbev/msq029 ; PubMed Central PMCID: PMCPMC2877997. 20118192 PMC2877997

[17] Alverson AJ, Zhuo S, Rice DW, Sloan DB, Palmer JD. The mitochondrial genome of the legume Vigna radiata and the analysis of recombination across short mitochondrial repeats. PLoS One. 2011;6(1):e16404 Epub 2011/02/02. 10.1371/journal.pone.0016404 ; PubMed Central PMCID: PMCPMC3024419. 21283772 PMC3024419

[18] Renner SS, Bellot S. Horizontal gene transfer in eukaryotes: fungi-to-plant and plant-to-plant transfers of organellar DNA Genomics of chloroplasts and mitochondria: Springer; 2012 p. 223–35.

[19] Sloan DB, Alverson AJ, Chuckalovcak JP, Wu M, McCauley DE, Palmer JD, et al Rapid evolution of enormous, multichromosomal genomes in flowering plant mitochondria with exceptionally high mutation rates. PLoS Biol. 2012;10(1):e1001241 Epub 2012/01/25. 10.1371/journal.pbio.1001241 ; PubMed Central PMCID: PMCPMC3260318. 22272183 PMC3260318

[20] Alverson AJ, Rice DW, Dickinson S, Barry K, Palmer JD. Origins and recombination of the bacterial-sized multichromosomal mitochondrial genome of cucumber. Plant Cell. 2011;23(7):2499–513. Epub 2011/07/12. 10.1105/tpc.111.087189 ; PubMed Central PMCID: PMCPMC3226218. 21742987 PMC3226218

[21] Leister D. Origin, evolution and genetic effects of nuclear insertions of organelle DNA. Trends Genet. 2005;21(12):655–63. Epub 2005/10/12. 10.1016/j.tig.2005.09.004 . 16216380

[22] Richly E, Leister D. NUPTs in sequenced eukaryotes and their genomic organization in relation to NUMTs. Mol Biol Evol. 2004;21(10):1972–80. Epub 2004/07/16. 10.1093/molbev/msh210 . 15254258

[23] Huang CY, Grunheit N, Ahmadinejad N, Timmis JN, Martin W. Mutational decay and age of chloroplast and mitochondrial genomes transferred recently to angiosperm nuclear chromosomes. Plant Physiol. 2005;138(3):1723–33. Epub 2005/06/14. 10.1104/pp.105.060327 ; PubMed Central PMCID: PMCPMC1176441. 15951485 PMC1176441

[24] Noutsos C, Richly E, Leister D. Generation and evolutionary fate of insertions of organelle DNA in the nuclear genomes of flowering plants. Genome Res. 2005;15(5):616–28. Epub 2005/05/04. 10.1101/gr.3788705 ; PubMed Central PMCID: PMCPMC1088290. 15867426 PMC1088290

[25] Lilly JW, Havey MJ. Small, repetitive DNAs contribute significantly to the expanded mitochondrial genome of cucumber. Genetics. 2001;159(1):317–28. WOS:000171252500026. 11560907 10.1093/genetics/159.1.317 PMC1461790

[26] Hazkani-Covo E, Zeller RM, Martin W. Molecular poltergeists: mitochondrial DNA copies (numts) in sequenced nuclear genomes. PLoS Genet. 2010;6(2):e1000834 Epub 2010/02/20. 10.1371/journal.pgen.1000834 ; PubMed Central PMCID: PMCPMC2820518. 20168995 PMC2820518

[27] Goremykin VV, Lockhart PJ, Viola R, Velasco R. The mitochondrial genome of Malus domestica and the import-driven hypothesis of mitochondrial genome expansion in seed plants. Plant J. 2012;71(4):615–26. Epub 2012/04/04. 10.1111/j.1365-313X.2012.05014.x . 22469001

[28] Notsu Y, Masood S, Nishikawa T, Kubo N, Akiduki G, Nakazono M, et al The complete sequence of the rice (Oryza sativa L.) mitochondrial genome: frequent DNA sequence acquisition and loss during the evolution of flowering plants. Mol Genet Genomics. 2002;268(4):434–45. Epub 2002/12/10. 10.1007/s00438-002-0767-1 . 12471441

[29] Clifton SW, Minx P, Fauron CM, Gibson M, Allen JO, Sun H, et al Sequence and comparative analysis of the maize NB mitochondrial genome. Plant Physiol. 2004;136(3):3486–503. Epub 2004/11/16. 10.1104/pp.104.044602 ; PubMed Central PMCID: PMCPMC527149. 15542500 PMC527149

[30] Sloan DB, Wu Z. History of plastid DNA insertions reveals weak deletion and at mutation biases in angiosperm mitochondrial genomes. Genome Biol Evol. 2014;6(12):3210–21. Epub 2014/11/25. 10.1093/gbe/evu253 ; PubMed Central PMCID: PMCPMC4986453. 25416619 PMC4986453

[31] Goremykin VV, Salamini F, Velasco R, Viola R. Mitochondrial DNA of Vitis vinifera and the issue of rampant horizontal gene transfer. Mol Biol Evol. 2009;26(1):99–110. Epub 2008/10/17. 10.1093/molbev/msn226 . 18922764

[32] Rodriguez-Moreno L, Gonzalez VM, Benjak A, Marti MC, Puigdomenech P, Aranda MA, et al Determination of the melon chloroplast and mitochondrial genome sequences reveals that the largest reported mitochondrial genome in plants contains a significant amount of DNA having a nuclear origin. BMC Genomics. 2011;12(1):424 Epub 2011/08/23. 10.1186/1471-2164-12-424 ; PubMed Central PMCID: PMCPMC3175227. 21854637 PMC3175227

[33] Gandini CL, Sanchez-Puerta MV. Foreign Plastid Sequences in Plant Mitochondria are Frequently Acquired Via Mitochondrion-to-Mitochondrion Horizontal Transfer. Sci Rep. 2017;7:43402 Epub 2017/03/07. 10.1038/srep43402 ; PubMed Central PMCID: PMCPMC5338292. 28262720 PMC5338292

[34] Wang XC, Chen H, Yang D, Liu C. Diversity of mitochondrial plastid DNAs (MTPTs) in seed plants. Mitochondrial DNA A DNA Mapp Seq Anal. 2017:1–8. Epub 2017/06/03. 10.1080/24701394.2017.1334772 . 28573928

[35] Wang D, Wu YW, Shih AC, Wu CS, Wang YN, Chaw SM. Transfer of chloroplast genomic DNA to mitochondrial genome occurred at least 300 MYA. Mol Biol Evol. 2007;24(9):2040–8. Epub 2007/07/05. 10.1093/molbev/msm133 . 17609537

[36] Iorizzo M, Senalik D, Szklarczyk M, Grzebelus D, Spooner D, Simon P. De novo assembly of the carrot mitochondrial genome using next generation sequencing of whole genomic DNA provides first evidence of DNA transfer into an angiosperm plastid genome. BMC Plant Biol. 2012;12(1):61 Epub 2012/05/03. 10.1186/1471-2229-12-61 ; PubMed Central PMCID: PMCPMC3413510. 22548759 PMC3413510

[37] Straub SC, Cronn RC, Edwards C, Fishbein M, Liston A. Horizontal transfer of DNA from the mitochondrial to the plastid genome and its subsequent evolution in milkweeds (apocynaceae). Genome Biol Evol. 2013;5(10):1872–85. Epub 2013/09/14. 10.1093/gbe/evt140 ; PubMed Central PMCID: PMCPMC3814198. 24029811 PMC3814198

[38] Smith DR. Mitochondrion-to-plastid DNA transfer: it happens. New Phytol. 2014;202(3):736–8. Epub 2014/01/29. 10.1111/nph.12704 . 24467712

[39] Downie SR, Jansen RK. A Comparative Analysis of Whole Plastid Genomes from the Apiales: Expansion and Contraction of the Inverted Repeat, Mitochondrial to Plastid Transfer of DNA, and Identification of Highly Divergent Noncoding Regions. Systematic Botany. 2015;40(1):336–51. 10.1600/036364415x686620 WOS:000350250100034.

[40] Ma PF, Zhang YX, Guo ZH, Li DZ. Evidence for horizontal transfer of mitochondrial DNA to the plastid genome in a bamboo genus. Sci Rep. 2015;5:11608 Epub 2015/06/24. 10.1038/srep11608 ; PubMed Central PMCID: PMCPMC4477325. 26100509 PMC4477325

[41] Chen JM, Chuzhanova N, Stenson PD, Férec C, Cooper DN. Meta‐Analysis of gross insertions causing human genetic disease: Novel mutational mechanisms and the role of replication slippage. Hum Mutat. 2005;25(2):207–21. 10.1002/humu.20133 15643617

[42] Sorenson MD, Quinn TW. Numts: A challenge for avian systematics and population biology. Auk. 1998;115(1):214–21. 10.2307/4089130 WOS:000071487100026.

[43] van der Kuyl AC, Kuiken CL, Dekker JT, Perizonius WR, Goudsmit J. Nuclear counterparts of the cytoplasmic mitochondrial 12S rRNA gene: a problem of ancient DNA and molecular phylogenies. J Mol Evol. 1995;40(6):652–7. . 7543951 10.1007/BF00160513

[44] Richly E, Leister D. NUMTs in sequenced eukaryotic genomes. Mol Biol Evol. 2004;21(6):1081–4. 10.1093/molbev/msh110 . 15014143

[45] Blanchard JL, Schmidt GW. Mitochondrial DNA migration events in yeast and humans: integration by a common end-joining mechanism and alternative perspectives on nucleotide substitution patterns. Mol Biol Evol. 1996;13(3):537–48. 10.1093/oxfordjournals.molbev.a025614 8742642

[46] Smith DR, Crosby K, Lee RW. Correlation between nuclear plastid DNA abundance and plastid number supports the limited transfer window hypothesis. Genome Biol Evol. 2011;3:365–71. 10.1093/gbe/evr001 21292629 PMC3101015

[47] Weese TL, Bohs L. A three-gene phylogeny of the genus Solanum (Solanaceae). Systematic Botany. 2007;32(2):445–63. 10.1600/036364407781179671 WOS:000247329000013.

[48] Consortium TG. The tomato genome sequence provides insights into fleshy fruit evolution. Nature. 2012;485(7400):635–41. 10.1038/nature11119 22660326 PMC3378239

[49] Consortium PGS. Genome sequence and analysis of the tuber crop potato. Nature. 2011;475(7355):189–95. 10.1038/nature10158 21743474

[50] Kim S, Park M, Yeom SI, Kim YM, Lee JM, Lee HA, et al Genome sequence of the hot pepper provides insights into the evolution of pungency in Capsicum species. Nat Genet. 2014;46(3):270–8. Epub 2014/01/21. 10.1038/ng.2877 . 24441736

[51] Chung HJ, Jung JD, Park HW, Kim JH, Cha HW, Min SR, et al The complete chloroplast genome sequences of Solanum tuberosum and comparative analysis with Solanaceae species identified the presence of a 241-bp deletion in cultivated potato chloroplast DNA sequence. Plant Cell Rep. 2006;25(12):1369–79. Epub 2006/07/13. 10.1007/s00299-006-0196-4 . 16835751

[52] Kahlau S, Aspinall S, Gray JC, Bock R. Sequence of the tomato chloroplast DNA and evolutionary comparison of solanaceous plastid genomes. J Mol Evol. 2006;63(2):194–207. Epub 2006/07/11. 10.1007/s00239-005-0254-5 . 16830097

[53] Daniell H, Lee SB, Grevich J, Saski C, Quesada-Vargas T, Guda C, et al Complete chloroplast genome sequences of Solanum bulbocastanum, Solanum lycopersicum and comparative analyses with other Solanaceae genomes. Theor Appl Genet. 2006;112(8):1503–18. Epub 2006/04/01. 10.1007/s00122-006-0254-x . 16575560

[54] Bolger A, Scossa F, Bolger ME, Lanz C, Maumus F, Tohge T, et al The genome of the stress-tolerant wild tomato species Solanum pennellii. Nat Genet. 2014;46(9):1034–8. Epub 2014/07/30. 10.1038/ng.3046 . 25064008 PMC7036041

[55] Wu Z. The completed eight chloroplast genomes of tomato from Solanum genus. Mitochondrial DNA A DNA Mapp Seq Anal. 2016;27(6):4155–7. Epub 2015/01/22. 10.3109/19401736.2014.1003890 . 25604480

[56] Cho KS, Park TH. Complete chloroplast genome sequence of Solanum nigrum and development of markers for the discrimination of S. nigrum. Hortic Environ Biote. 2016;57(1):69–78. 10.1007/s13580-016-0003-2 WOS:000371261800009.

[57] Cho KS, Cheon KS, Hong SY, Cho JH, Im JS, Mekapogu M, et al Complete chloroplast genome sequences of Solanum commersonii and its application to chloroplast genotype in somatic hybrids with Solanum tuberosum. Plant Cell Rep. 2016;35(10):2113–23. Epub 2016/07/16. 10.1007/s00299-016-2022-y . 27417695

[58] Shikanai T, Kaneko H, Nakata S, Harada K, Watanabe K. Mitochondrial genome structure of a cytoplasmic hybrid between tomato and wild potato. Plant Cell Reports. 1998;17(11):832–6. 10.1007/s002990050493 WOS:000075338000002. 30736552

[59] Tomato Genome Sequencing C, Aflitos S, Schijlen E, de Jong H, de Ridder D, Smit S, et al Exploring genetic variation in the tomato (Solanum section Lycopersicon) clade by whole-genome sequencing. Plant J. 2014;80(1):136–48. Epub 2014/07/22. 10.1111/tpj.12616 . 25039268

[60] Kearse M, Moir R, Wilson A, Stones-Havas S, Cheung M, Sturrock S, et al Geneious Basic: an integrated and extendable desktop software platform for the organization and analysis of sequence data. Bioinformatics. 2012;28(12):1647–9. Epub 2012/05/01. 10.1093/bioinformatics/bts199 ; PubMed Central PMCID: PMCPMC3371832. 22543367 PMC3371832

[61] Hahn C, Bachmann L, Chevreux B. Reconstructing mitochondrial genomes directly from genomic next-generation sequencing reads—a baiting and iterative mapping approach. Nucleic Acids Res. 2013;41(13):e129 Epub 2013/05/11. 10.1093/nar/gkt371 ; PubMed Central PMCID: PMCPMC3711436. 23661685 PMC3711436

[62] Kim HT, Chase MW. Independent degradation in genes of the plastid ndh gene family in species of the orchid genus Cymbidium (Orchidaceae; Epidendroideae). PLoS One. 2017;12(11):e0187318 Epub 2017/11/16. 10.1371/journal.pone.0187318 ; PubMed Central PMCID: PMCPMC5695243. 29140976 PMC5695243

[63] Li H, Durbin R. Fast and accurate short read alignment with Burrows-Wheeler transform. Bioinformatics. 2009;25(14):1754–60. Epub 2009/05/20. 10.1093/bioinformatics/btp324 ; PubMed Central PMCID: PMCPMC2705234. 19451168 PMC2705234

[64] Kim HT, Kim JS, Moore MJ, Neubig KM, Williams NH, Whitten WM, et al Seven New Complete Plastome Sequences Reveal Rampant Independent Loss of the ndh Gene Family across Orchids and Associated Instability of the Inverted Repeat/Small Single-Copy Region Boundaries. PLoS One. 2015;10(11):e0142215 Epub 2015/11/13. 10.1371/journal.pone.0142215 ; PubMed Central PMCID: PMCPMC4641739. 26558895 PMC4641739

[65] Altschul SF, Gish W, Miller W, Myers EW, Lipman DJ. Basic local alignment search tool. J Mol Biol. 1990;215(3):403–10. Epub 1990/10/05. 10.1016/S0022-2836(05)80360-2 . 2231712

[66] Lowe TM, Eddy SR. tRNAscan-SE: a program for improved detection of transfer RNA genes in genomic sequence. Nucleic Acids Res. 1997;25(5):955–64. Epub 1997/03/01. ; PubMed Central PMCID: PMCPMC146525. 9023104 10.1093/nar/25.5.955 PMC146525

[67] Darzentas N. Circoletto: visualizing sequence similarity with Circos. Bioinformatics. 2010;26(20):2620–1. Epub 2010/08/26. 10.1093/bioinformatics/btq484 . 20736339

[68] Ihaka R, Gentleman R. R: a language for data analysis and graphics. Journal of computational and graphical statistics. 1996;5(3):299–314.

[69] Wickham H. ggplot2: elegant graphics for data analysis: Springer; 2016.

[70] Auguie B. gridExtra: functions in Grid graphics. R package version 09. 2012;1.

[71] Guy L, Kultima JR, Andersson SG. genoPlotR: comparative gene and genome visualization in R. Bioinformatics. 2010;26(18):2334–5. Epub 2010/07/14. 10.1093/bioinformatics/btq413 ; PubMed Central PMCID: PMCPMC2935412. 20624783 PMC2935412

[72] Lohse M, Drechsel O, Bock R. OrganellarGenomeDRAW (OGDRAW): a tool for the easy generation of high-quality custom graphical maps of plastid and mitochondrial genomes. Curr Genet. 2007;52(5–6):267–74. Epub 2007/10/25. 10.1007/s00294-007-0161-y . 17957369

[73] Lichtenstein G, Conte M, Asis R, Carrari F. Chloroplast and Mitochondrial Genomes of Tomato The Tomato Genome: Springer; 2016 p. 111–37.

[74] Group AP. An update of the Angiosperm Phylogeny Group classification for the orders and families of flowering plants: APG III. Bot J Linn Soc. 2009;161(2):105–21.

[75] Olmstead RG, Bohs L, Migid HA, Santiago-Valentin E, Garcia VF, Collier SM. A molecular phylogeny of the Solanaceae. Taxon. 2008;57(4):1159–81. WOS:000261283000010.

[76] Van de Paer C, Hong-Wa C, Jeziorski C, Besnard G. Mitogenomics of Hesperelaea, an extinct genus of Oleaceae. Gene. 2016;594(2):197–202. Epub 2016/09/08. 10.1016/j.gene.2016.09.007 . 27601255

[77] Knoop V. The mitochondrial DNA of land plants: peculiarities in phylogenetic perspective. Curr Genet. 2004;46(3):123–39. Epub 2004/08/10. 10.1007/s00294-004-0522-8 . 15300404

[78] Jo YD, Choi Y, Kim DH, Kim BD, Kang BC. Extensive structural variations between mitochondrial genomes of CMS and normal peppers (Capsicum annuum L.) revealed by complete nucleotide sequencing. BMC Genomics. 2014;15:561 Epub 2014/07/06. 10.1186/1471-2164-15-561 ; PubMed Central PMCID: PMCPMC4108787. 24996600 PMC4108787

[79] Chang S, Wang Y, Lu J, Gai J, Li J, Chu P, et al The mitochondrial genome of soybean reveals complex genome structures and gene evolution at intercellular and phylogenetic levels. PLoS One. 2013;8(2):e56502 Epub 2013/02/23. 10.1371/journal.pone.0056502 ; PubMed Central PMCID: PMCPMC3576410. 23431381 PMC3576410

[80] Gao C, Ren X, Mason AS, Liu H, Xiao M, Li J, et al Horizontal gene transfer in plants. Funct Integr Genomics. 2014;14(1):23–9. Epub 2013/10/18. 10.1007/s10142-013-0345-0 . 24132513

[81] Rice DW, Alverson AJ, Richardson AO, Young GJ, Sanchez-Puerta MV, Munzinger J, et al Horizontal transfer of entire genomes via mitochondrial fusion in the angiosperm Amborella. Science. 2013;342(6165):1468–73. Epub 2013/12/21. 10.1126/science.1246275 . 24357311

[82] Chaw SM, Shih AC, Wang D, Wu YW, Liu SM, Chou TY. The mitochondrial genome of the gymnosperm Cycas taitungensis contains a novel family of short interspersed elements, Bpu sequences, and abundant RNA editing sites. Mol Biol Evol. 2008;25(3):603–15. Epub 2008/01/15. 10.1093/molbev/msn009 . 18192697

[83] Lonsdale DM, Hodge TP, Fauron CM. The physical map and organisation of the mitochondrial genome from the fertile cytoplasm of maize. Nucleic Acids Res. 1984;12(24):9249–61. Epub 1984/12/21. ; PubMed Central PMCID: PMCPMC320458. 6096824 10.1093/nar/12.24.9249 PMC320458

[84] Tanaka Y, Tsuda M, Yasumoto K, Yamagishi H, Terachi T. A complete mitochondrial genome sequence of Ogura-type male-sterile cytoplasm and its comparative analysis with that of normal cytoplasm in radish (Raphanus sativus L.). BMC Genomics. 2012;13(1):352 Epub 2012/08/01. 10.1186/1471-2164-13-352 ; PubMed Central PMCID: PMCPMC3473294. 22846596 PMC3473294

[85] Sloan DB, Muller K, McCauley DE, Taylor DR, Storchova H. Intraspecific variation in mitochondrial genome sequence, structure, and gene content in Silene vulgaris, an angiosperm with pervasive cytoplasmic male sterility. New Phytol. 2012;196(4):1228–39. Epub 2012/09/27. 10.1111/j.1469-8137.2012.04340.x . 23009072

[86] Allen JO, Fauron CM, Minx P, Roark L, Oddiraju S, Lin GN, et al Comparisons among two fertile and three male-sterile mitochondrial genomes of maize. Genetics. 2007;177(2):1173–92. Epub 2007/07/31. 10.1534/genetics.107.073312 ; PubMed Central PMCID: PMCPMC2034622. 17660568 PMC2034622

[87] Darracq A, Varre JS, Marechal-Drouard L, Courseaux A, Castric V, Saumitou-Laprade P, et al Structural and content diversity of mitochondrial genome in beet: a comparative genomic analysis. Genome Biol Evol. 2011;3:723–36. Epub 2011/05/24. 10.1093/gbe/evr042 ; PubMed Central PMCID: PMCPMC3163473. 21602571 PMC3163473

[88] Palmer JD, Shields CR. Tripartite structure of the Brassica campestris mitochondrial genome. Nature. 1984;307(5950):437–40.

[89] Sarkinen T, Bohs L, Olmstead RG, Knapp S. A phylogenetic framework for evolutionary study of the nightshades (Solanaceae): a dated 1000-tip tree. BMC Evol Biol. 2013;13(1):214 Epub 2013/11/29. 10.1186/1471-2148-13-214 ; PubMed Central PMCID: PMCPMC3850475. 24283922 PMC3850475

[90] Michalovova M, Vyskot B, Kejnovsky E. Analysis of plastid and mitochondrial DNA insertions in the nucleus (NUPTs and NUMTs) of six plant species: size, relative age and chromosomal localization. Heredity (Edinb). 2013;111(4):314–20. Epub 2013/05/30. 10.1038/hdy.2013.51 ; PubMed Central PMCID: PMCPMC3807264. 23715017 PMC3807264

[91] Kleine T, Maier UG, Leister D. DNA transfer from organelles to the nucleus: the idiosyncratic genetics of endosymbiosis. Annu Rev Plant Biol. 2009;60:115–38. Epub 2008/11/19. 10.1146/annurev.arplant.043008.092119 . 19014347

