# Supplementary figures and images for: Organellar genome analysis reveals endosymbiotic gene transfers in tomato
Source: PLoS One. 2018 Sep 5;13(9):e0202279. doi: 10.1371/journal.pone.0202279 (PMC6124701; doi:10.1371/journal.pone.0202279)

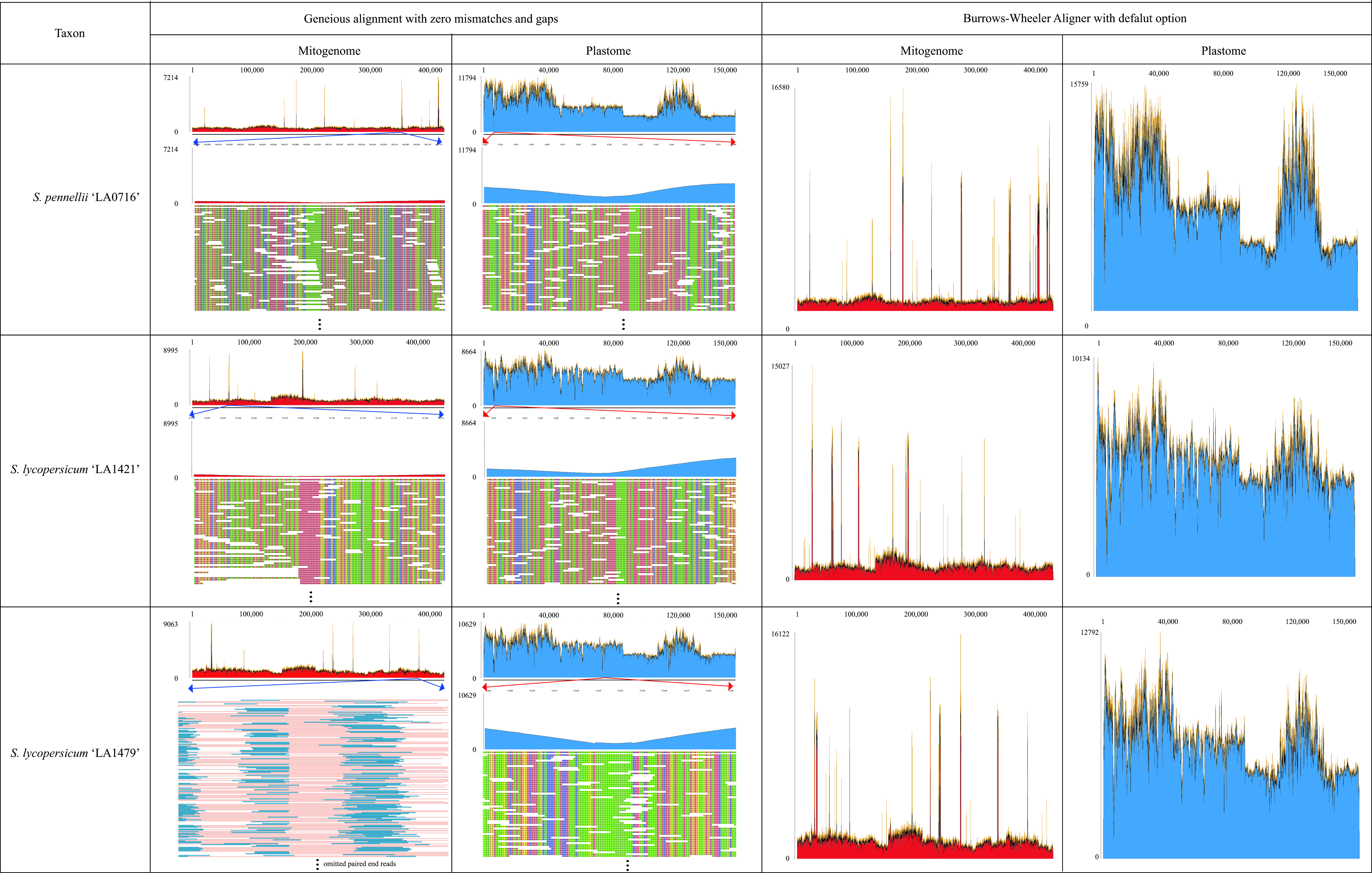

Supplement: S1 Fig — Raw reads were mapped to mitogenomes and plastomes using Geneious aligner with zero mismatch and gap among the reads, and the Burrows-Wheeler alignment tool with the default options set to verify the coverage depths through the genome. Sharp peaks that were up to 20-fold higher than base coverage indicate mitochondrial plastome regions. Coverages were higher than 200, except for certain regions containing homopolymers or AT-rich regions, which had low coverage depth. However, these regions were also supported by numerous paired-end reads (blue bar and red line indicate paired-end reads and intervals between paired-end reads, respectively, in Solanum lycopersicum ‘LA1479’). The X-axis and Y-axis indicate positions and coverage depths, respectively. (TIF) [file pone.0202279.s001.tif]

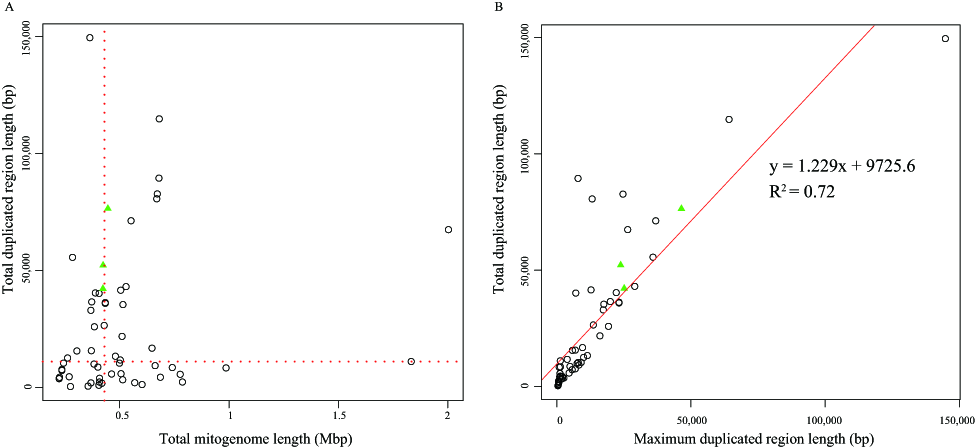

Supplement: S2 Fig — (A) Total mitogenome length vs total duplicated region length. (B) Maximum lengths of duplicated regions vs total lengths of duplicated regions. Green triangles represent the three tomato mitogenomes. (TIF) [file pone.0202279.s002.tif]

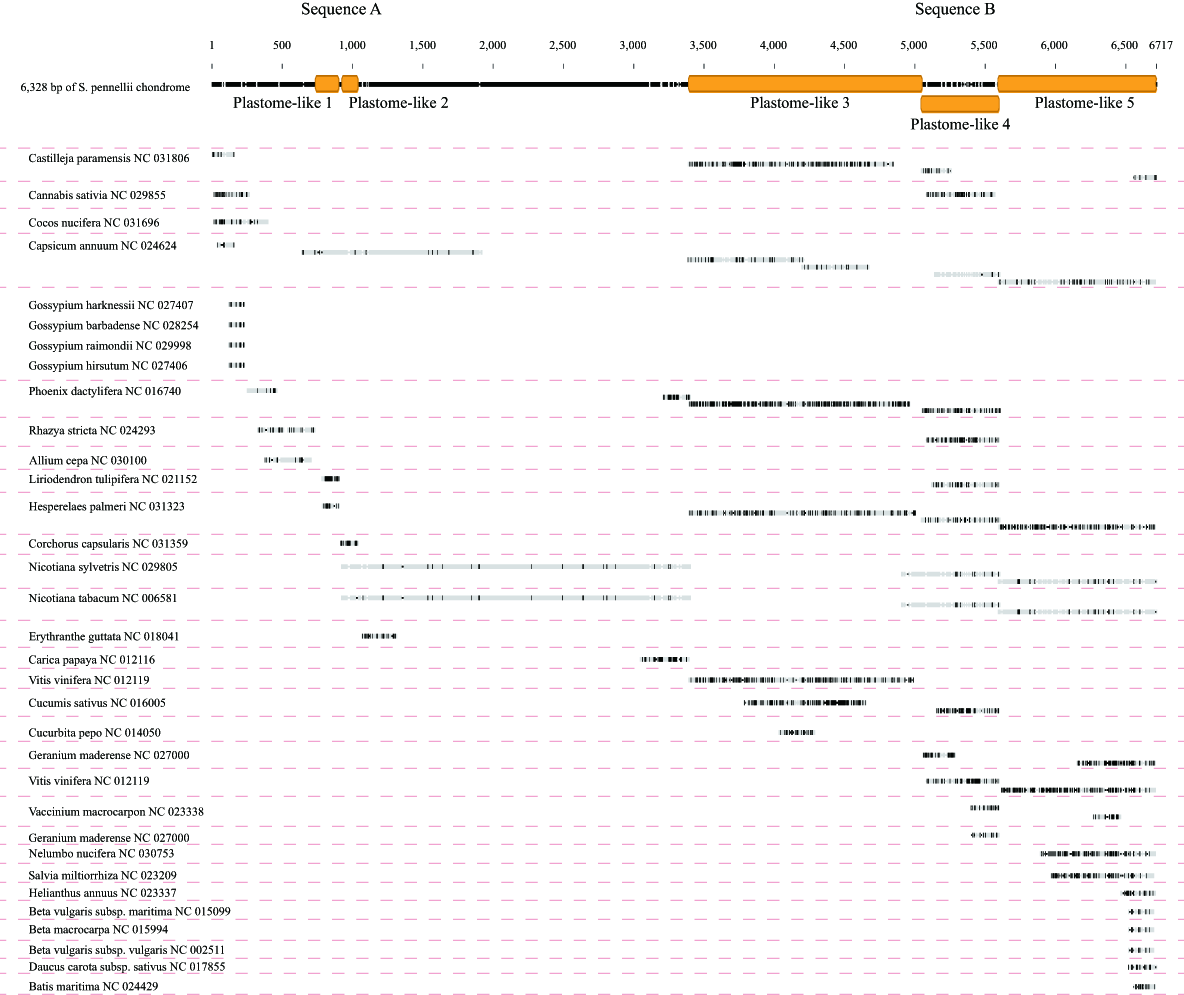

Supplement: S3 Fig — Species are divided by dashed red lines. The yellow box on top represents mitochondrial plastid DNAs. The gray regions on the other angiosperm chromosomes are more similar to the S. pennellii ‘LA0716’ mitogenome than the black regions. (TIF) [file pone.0202279.s003.tif]

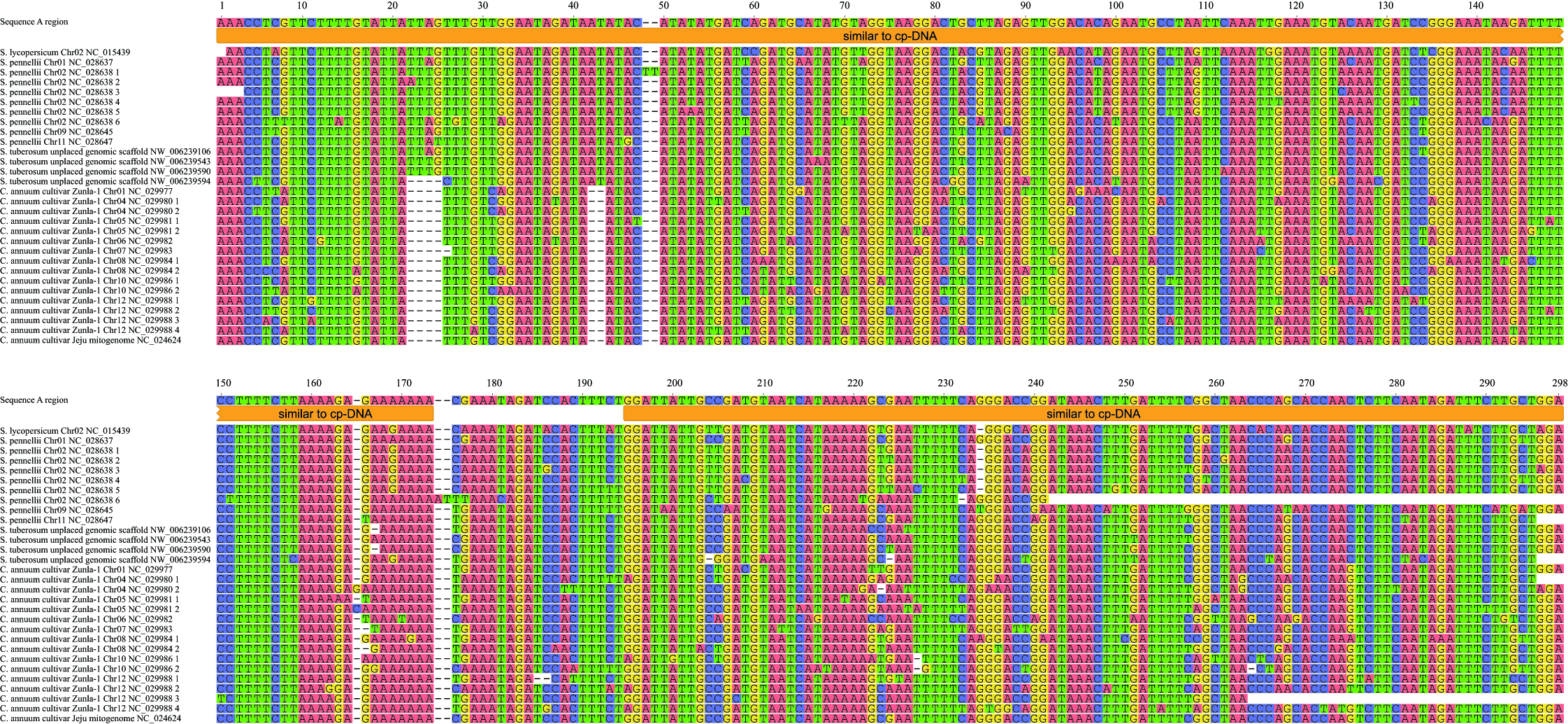

Supplement: S4 Fig — Numerous sequence-A-similar regions were observed in the S. pennellii, S. tuberosum, and C. annuum nuclear genomes; however, one sequence A copy was also observed in the S. lycopersicum ‘Heinz1706’ nuclear genome. (TIF) [file pone.0202279.s004.tif]

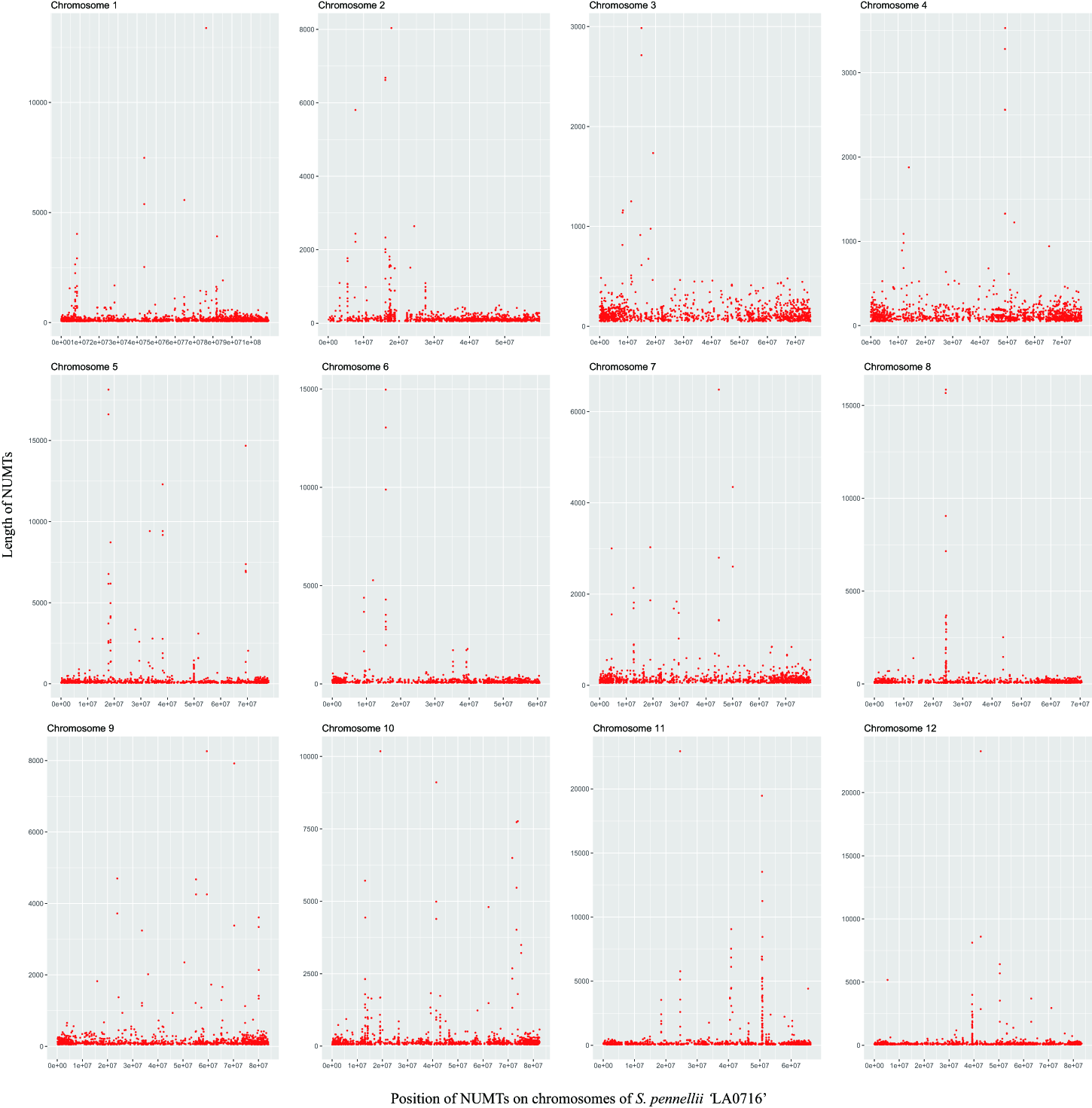

Supplement: S5 Fig — The X-axis indicates the positions of the NUMTs and the Y-axis indicates the lengths of the NUMTs on each chromosome of S. pennellii ‘LA0716’. (TIF) [file pone.0202279.s005.tif]

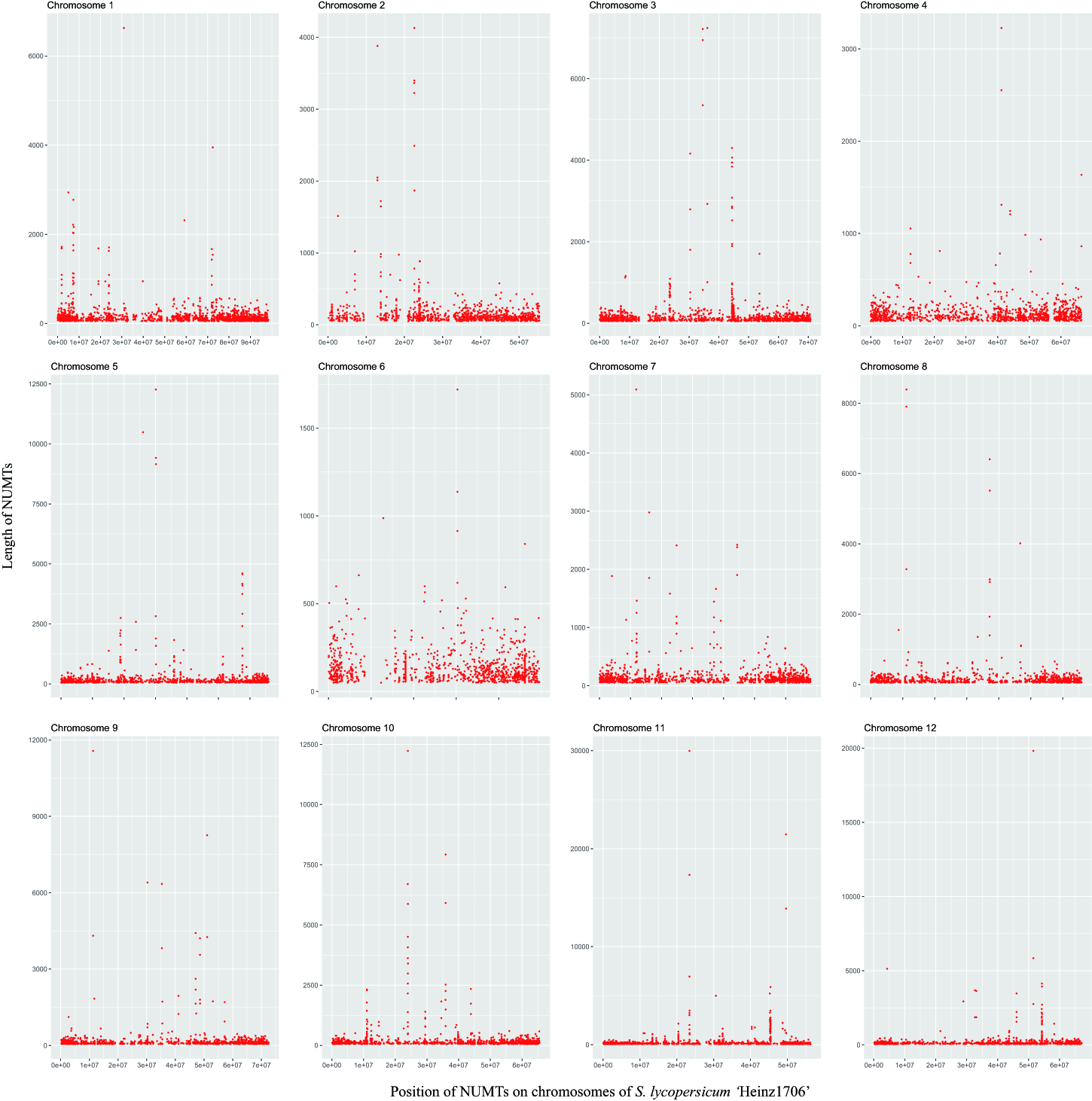

Supplement: S6 Fig — The X-axis indicates the positions of the NUMTs and the Y-axis indicates the lengths of the NUMTs on each chromosome of S. lycopersicum ‘Heinz1706’. (TIF) [file pone.0202279.s006.tif]

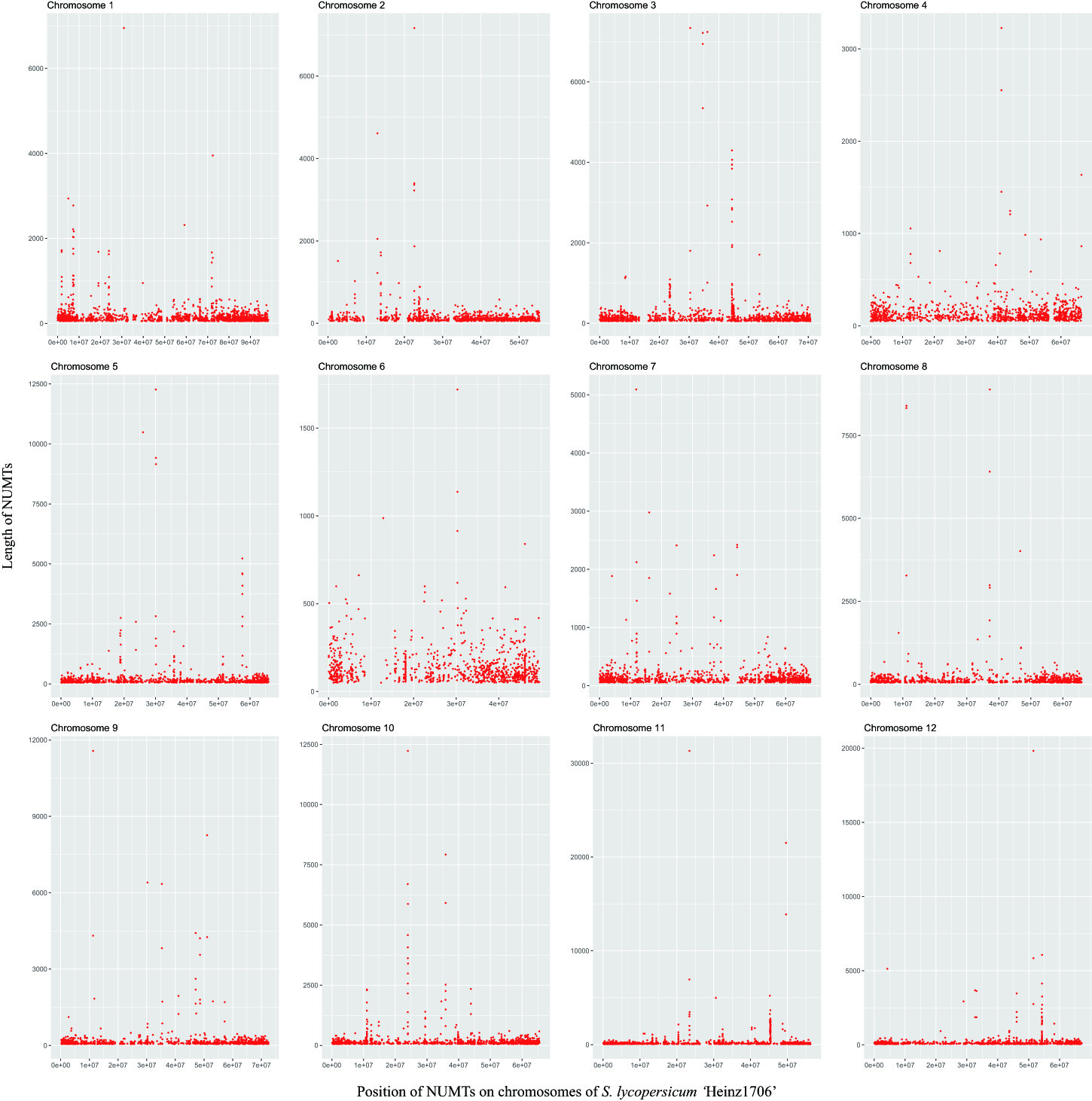

Supplement: S7 Fig — The X-axis indicates the positions of the NUMTs and the Y-axis indicates the lengths of the NUMTs on each chromosome of S. lycopersicum ‘Heinz1706’. (TIF) [file pone.0202279.s007.tif]

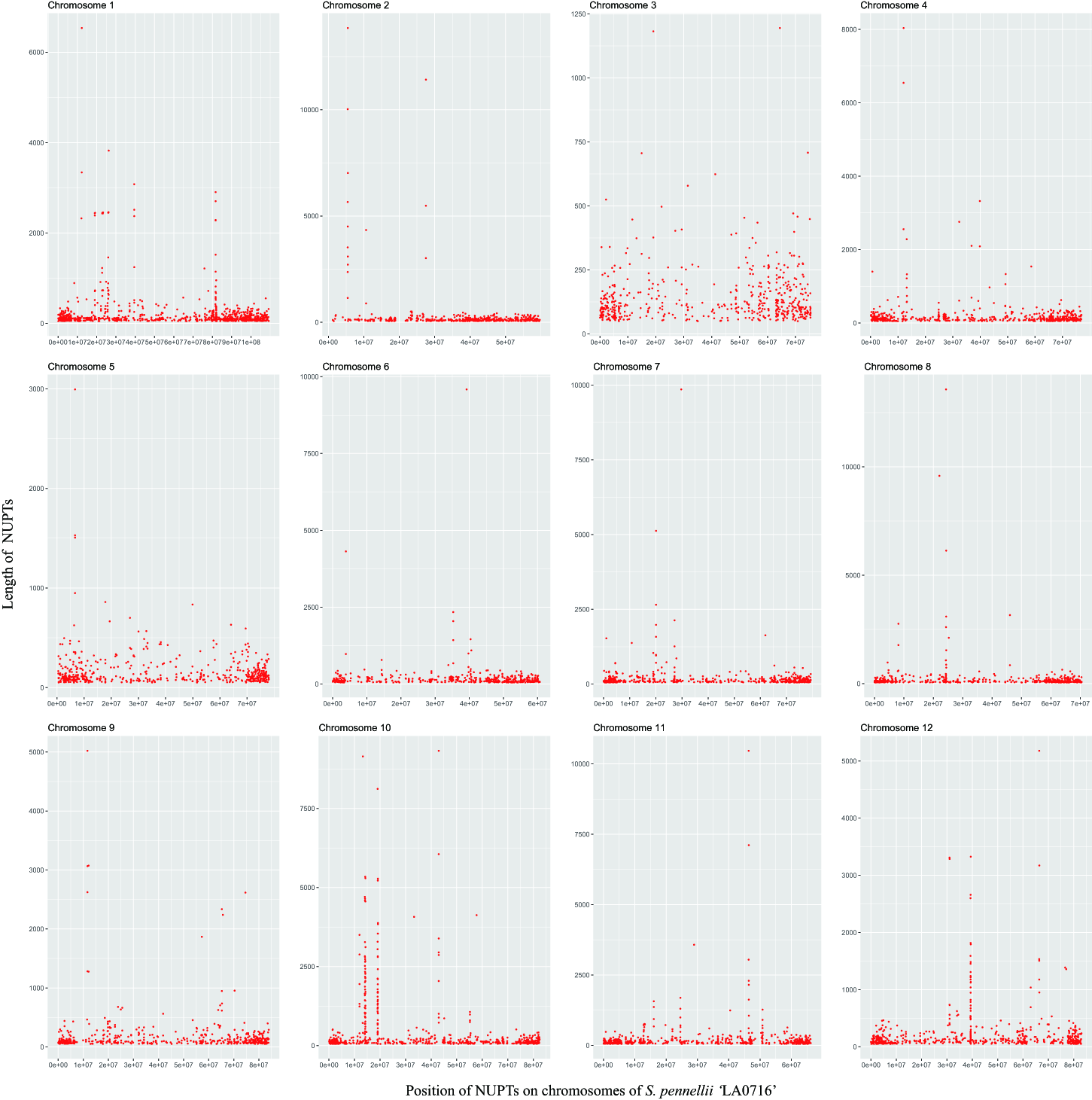

Supplement: S8 Fig — The X-axis indicates the positions of the NUPTs and the Y-axis indicates the lengths of the NUPTs on each chromosome of S. pennellii ‘LA0716’. (TIF) [file pone.0202279.s008.tif]

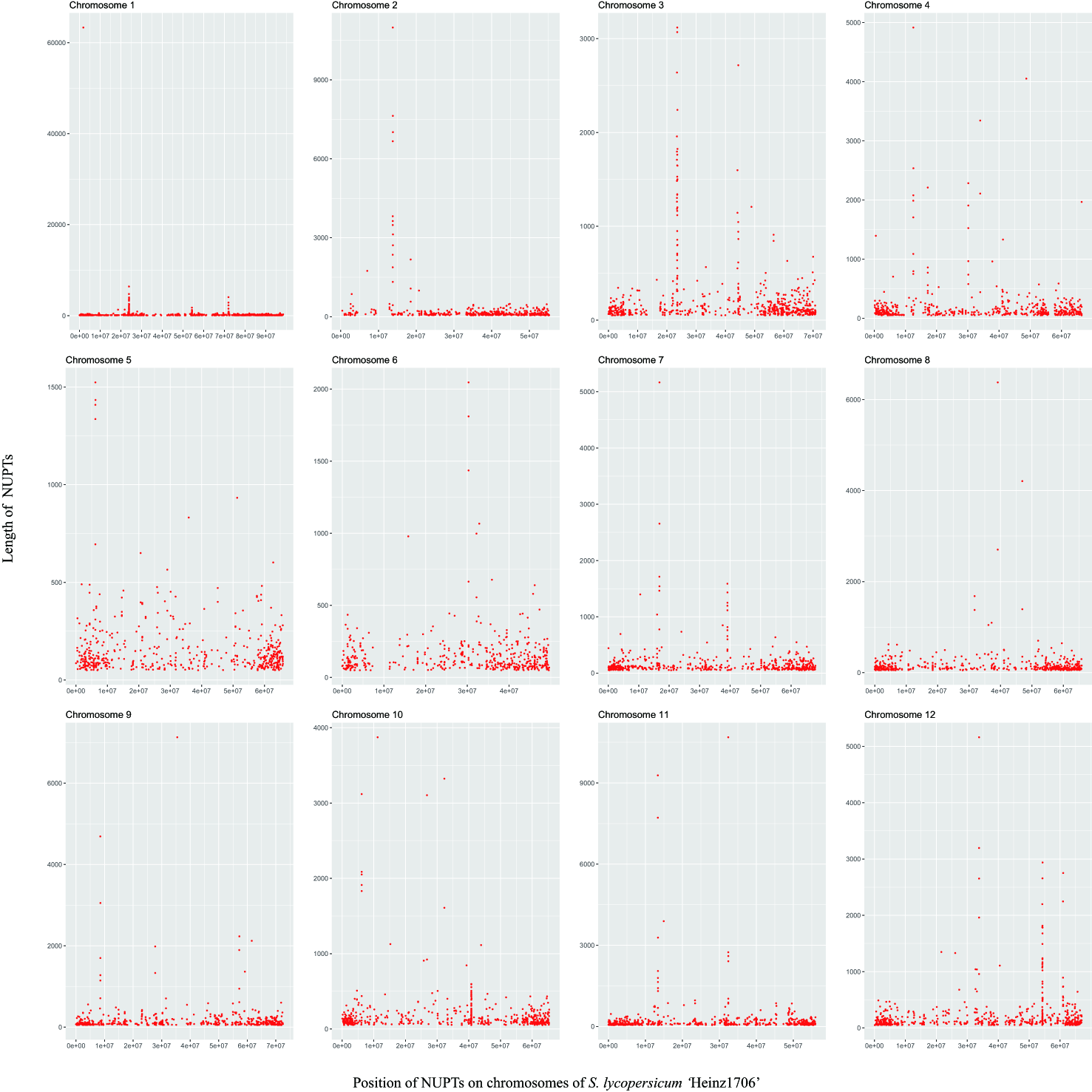

Supplement: S9 Fig — The X-axis indicates the positions of the NUPTs and the Y-axis indicates the lengths of the NUPTs on each chromosome of S. lycopersicum ‘Heinz1706’. (TIF) [file pone.0202279.s009.tif]

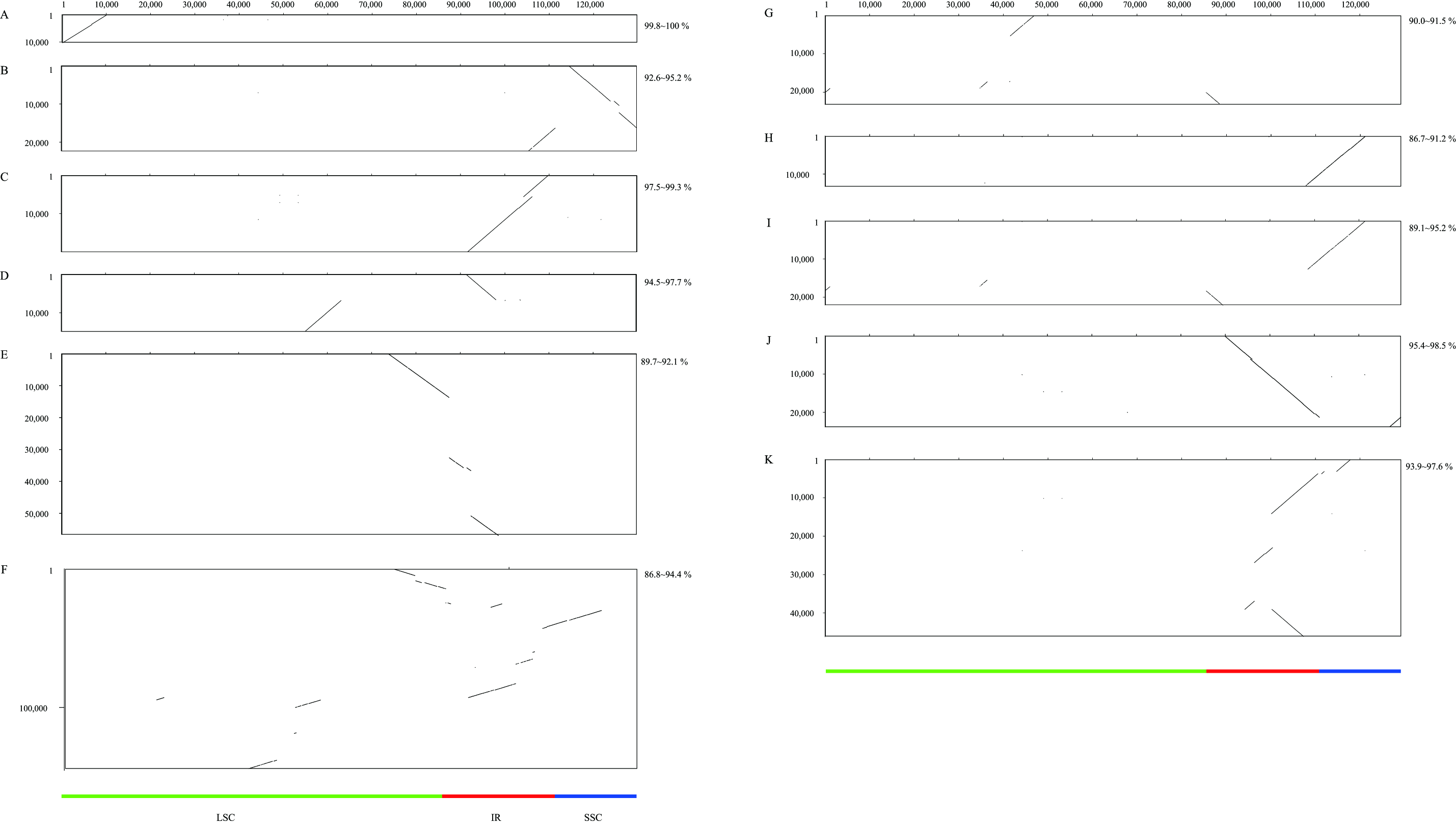

Supplement: S10 Fig — The percentages located on the right-hand side of the boxes indicate the similarity between NUPTs (≥1,000 bp) and their counterparts in the plastome. The colored line at the bottom indicates the positions of large single copy (LSC), inverted repeat (IR), and small single copy (SSC) regions. (TIF) [file pone.0202279.s010.tif]

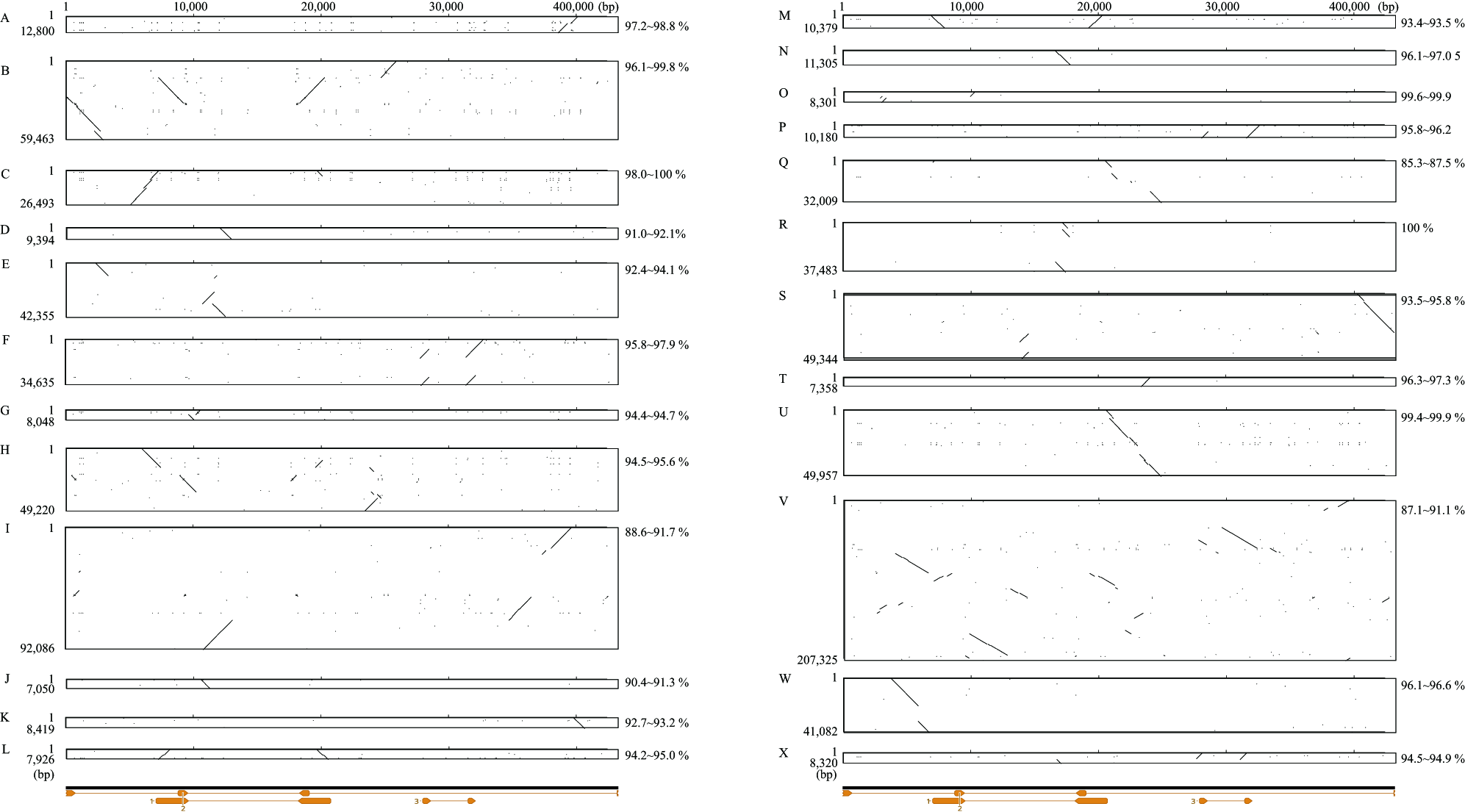

Supplement: S11 Fig — The percentages located on the right-hand side of the boxes indicate the similarity between NUMTs (≥1,000 bp) and their counterparts in the mitogenome. The colored arrow below the bottom line indicates large repeat sequences (≥5,000 bp) in the mitogenome. (TIF) [file pone.0202279.s011.tif]
