# Supplementary material for: Organellar genome analysis reveals endosymbiotic gene transfers in tomato
Source: PLoS One. 2018 Sep 5;13(9):e0202279. doi: 10.1371/journal.pone.0202279 (PMC6124701; doi:10.1371/journal.pone.0202279)
Supplement: S1 Table — (DOCX) [file pone.0202279.s012.docx]

**S1 Table. Mitogenome sequences in core eudicots.**

| Order | Family | Species | Accession | Order | Family | Species | Accession |
| --- | --- | --- | --- | --- | --- | --- | --- |
| Vitales | Vitaceae | *Vitis vinifera* | NC_012119 | Brassicales | Brassicaceae | *Brassica oleracea* | NC_016118 |
| Malpighiales | Euphorbiaceae | *Ricinus communis* | NC_015141 | Brassicales | Brassicaceae | *Brassica rapa* subsp*. oleifera* | NC_016125 |
| Malpighiales | Salicaceae | *Populus tremula* | NC_028096 | Brassicales | Brassicaceae | *Raphanus sativus* | NC_018551 |
| Malpighiales | Salicaceae | *Populus tremula* x *Populus alba* | NC_028329 | Brassicales | Brassicaceae | *Sinapis arvensis* | NC_031896 |
| Malpighiales | Salicaceae | *Salix purpurea* | NC_029693 | Brassicales | Caricaceae | *Carica papaya* | NC_012116 |
| Malpighiales | Salicaceae | *Salix suchowensis* | NC_029317 | Santalales | Viscaceae | *Viscum album* | NC_029039 |
| Fabales | Fabaceae | *Glycine max* | NC_020455 | Caryophyllales | Chenopodiaceae | *Beta macrocarpa* | NC_015994 |
| Fabales | Fabaceae | *Lotus japonicus* | NC_016743 | Caryophyllales | Chenopodiaceae | *Beta vulgaris* subsp*. maritima* | NC_015099 |
| Fabales | Fabaceae | *Medicago truncatula* | NC_029641 | Caryophyllales | Chenopodiaceae | *Beta vulgaris* subsp*. vulgaris* | NC_002511 |
| Fabales | Fabaceae | *Millettia pinnata* | NC_016742 | Ericales | Ericaceae | *Vaccinium macrocarpon* | NC_023338 |
| Fabales | Fabaceae | *Vigna angularis* | NC_021092 | Gentianales | Apocynaceae | *Asclepias syriaca* | NC_022796 |
| Fabales | Fabaceae | *Vigna radiata* | NC_015121 | Gentianales | Apocynaceae | *Rhazya stricta* | NC_024293 |
| Rosales | Cannabaceae | *Cannabis sativa* | NC_029855 | Solanales | Convolvulaceae | *Ipomoea nil* | NC_031158 |
| Rosales | Rhamnaceae | *Ziziphus jujuba* | NC_029809 | Solanales | Solanaceae | *Capsicum annuum* | NC_024624 |
| Rosales | Rosaceae | *Malus domestica* | NC_018554 | Solanales | Solanaceae | *Capsicum annuum* CMS | KJ865409 |
| Cucurbitales | Cucurbitaceae | *Citrullus lanatus* | NC_014043 | Solanales | Solanaceae | *Hyoscyamus niger* | NC_026515 |
| Cucurbitales | Cucurbitaceae | *Cucurbita pepo* | NC_014050 | Solanales | Solanaceae | *Nicotiana sylvestris* | NC_029805 |
| Geraniales | Geraniaceae | *Geranium maderense* | NC_027000 | Solanales | Solanaceae | *Nicotiana tabacum* | NC_006581 |
| Malvales | Malvaceae | *Corchorus capsularis* | NC_031359 | Solanales | Solanaceae | *S. lycopersicum* ‘LA1421’ | NC_035963 |
| Malvales | Malvaceae | *Corchorus olitorius* | NC_031360 | Solanales | Solanaceae | *S. lycopersicum* ‘LA1479’ | MF034193 |
| Malvales | Malvaceae | *Gossypium barbadense* | NC_028254 | Solanales | Solanaceae | *S. pennellii* ‘LA0716’ | NC_035964 |
| Malvales | Malvaceae | *Gossypium harknessii* | NC_027407 | Lamiales | Gesneriaceae | *Dorcoceras hygrometricum* | NC_016741 |
| Malvales | Malvaceae | *Gossypium hirsutum* | NC_027406 | Lamiales | Lamiaceae | *Ajuga reptans* | NC_023103 |
| Malvales | Malvaceae | *Gossypium raimondii* | NC_029998 | Lamiales | Lamiaceae | *Castilleja paramensis* | NC_023103 |
| Brassicales | Bataceae | *Batis maritima* | NC_024429 | Lamiales | Lamiaceae | *Salvia miltiorrhiza* | NC_023209 |
| Brassicales | Brassicaceae | *Arabidopsis thaliana* | NC_001284 | Lamiales | Oleaceae | *Hesperelaea palmeri* | NC_031323 |
| Brassicales | Brassicaceae | *Brassica carinata* | NC_016120 | Lamiales | Orobanchaceae | *Erythranthe guttata* | NC_031806 |
| Brassicales | Brassicaceae | *Brassica juncea* | NC_016123 | Asterales | Asteraceae | *Helianthus annuus* | NC_023337 |
| Brassicales | Brassicaceae | *Brassica napus* | NC_008285 | Apiales | Apiaceae | *Daucus carota* subsp*. sativus* | NC_017855 |
| Brassicales | Brassicaceae | *Brassica nigra* | NC_029182 |  |  |  |  |
