# Supplementary material for: Organellar genome analysis reveals endosymbiotic gene transfers in tomato
Source: PLoS One. 2018 Sep 5;13(9):e0202279. doi: 10.1371/journal.pone.0202279 (PMC6124701; doi:10.1371/journal.pone.0202279)
Supplement: S2 Table — (DOCX) [file pone.0202279.s013.docx]

**S2 Table. Regions with less than 200 coverage depths in the three mitogenomes.**

| Taxa | Geneious alignment with  zero mismatches and gaps | | | Burrows-Wheeler Aligner  with default option | | |
| --- | --- | --- | --- | --- | --- | --- |
|  | Start | End | Length | Start | End | Length |
| *S. pennellii* ‘LA0716’ | 8756 | 8781 | 26 | 78328 | 78343 | 16 |
|  | 8786 | 8786 | 1 | 198735 | 198748 | 14 |
|  | 22565 | 22566 | 2 | 348494 | 348495 | 2 |
|  | 40516 | 40516 | 1 | 355292 | 355366 | 75 |
|  | 70782 | 70788 | 7 |  |  |  |
|  | 70790 | 70791 | 2 |  |  |  |
|  | 70793 | 70793 | 1 |  |  |  |
|  | 77255 | 77282 | 28 |  |  |  |
|  | 85621 | 85623 | 3 |  |  |  |
|  | 102951 | 102955 | 5 |  |  |  |
|  | 161448 | 161466 | 19 |  |  |  |
|  | 164114 | 164121 | 8 |  |  |  |
|  | 164138 | 164145 | 8 |  |  |  |
|  | 181857 | 181858 | 2 |  |  |  |
|  | 195742 | 195770 | 29 |  |  |  |
|  | 195908 | 195915 | 8 |  |  |  |
|  | 262344 | 262362 | 19 |  |  |  |
|  | 271998 | 272000 | 3 |  |  |  |
|  | 272183 | 272196 | 14 |  |  |  |
|  | 308046 | 308047 | 2 |  |  |  |
|  | 308049 | 308049 | 1 |  |  |  |
|  | 308051 | 308051 | 1 |  |  |  |
|  | 316830 | 316854 | 25 |  |  |  |
|  | 318133 | 318136 | 4 |  |  |  |
|  | 318143 | 318147 | 5 |  |  |  |
|  | 343265 | 343305 | 41 |  |  |  |
|  | 349920 | 350048 | 129 |  |  |  |
|  | Minimum depth = 60 | | | Minimum depth = 83 | | |
| *S. lycopersicum* ‘LA1421’ | 9520 | 9541 | 22 | 60051 | 60079 | 29 |
|  | 9544 | 9557 | 14 | 68176 | 68246 | 71 |
|  | 26526 | 26533 | 8 |  |  |  |
|  | 26538 | 26570 | 33 |  |  |  |
|  | 51265 | 51296 | 32 |  |  |  |
|  | 59119 | 59161 | 43 |  |  |  |
|  | 67040 | 67133 | 94 |  |  |  |
|  | 122089 | 122104 | 16 |  |  |  |
|  | 122109 | 122113 | 5 |  |  |  |
|  | 122119 | 122127 | 9 |  |  |  |
|  | 243432 | 243437 | 6 |  |  |  |
|  | 243440 | 243441 | 2 |  |  |  |
|  | 245049 | 245054 | 6 |  |  |  |
|  | 400259 | 400259 | 1 |  |  |  |
|  | 400267 | 400276 | 10 |  |  |  |
|  | 401873 | 401878 | 6 |  |  |  |
|  | 401880 | 401880 | 1 |  |  |  |
|  | 401882 | 401891 | 10 |  |  |  |
|  | Minimum depth = 43 | | | Minimum depth = 82 | | |
| *S. lycopersicum* ‘LA1479’ | 234218 | 234267 | 50 | 237724 | 237751 | 28 |
|  | 242162 | 242188 | 27 | 245898 | 245921 | 24 |
|  | 378566 | 378605 | 40 |  |  |  |
|  | Minimum depth = 24 | | | Minimum depth = ­94 | | |
