# Supplementary material for: Organellar genome analysis reveals endosymbiotic gene transfers in tomato
Source: PLoS One. 2018 Sep 5;13(9):e0202279. doi: 10.1371/journal.pone.0202279 (PMC6124701; doi:10.1371/journal.pone.0202279)
Supplement: S3 Table — (DOCX) [file pone.0202279.s014.docx]

**S3 Table. Syntenic blocks in the three tomato mitogenomes.**

| Name | *S. pennellii* ‘LA0716’ | | | *S. lycopersicum* ‘LA1421’ | | | *S. lycopersicum* ‘LA1479’ | | | Gene content |
| --- | --- | --- | --- | --- | --- | --- | --- | --- | --- | --- |
|  | Start^a^ | End^b^ | Length^c^ | Start | End | Length | Start | End | Length |  |
| Syntenic block1 | 1 | 7456 | 7456 | 245302 | 252757 | 7456 | 152225 | 159680 | 7456 | *trnE-*TTC |
| Syntenic block2 | 7339 | 68551 | 61213 | 313895 | 375169 | 61275 | 73583 | 134828 | 61246 | *rps10, cox1, ccmFN, nad1* exon1*,nad1* exon2*, nad1* exon3*, trnI-*CAT*, nad7* |
| Syntenic block3 | 68775 | 93613 | 24839 | 375183 | 400021 | 24839 | 134842 | 159680 | 24839 | *rps13, nad1 exon4, rrn18, rrn5, trnE-*TTC |
| Syntenic block4 | 93496 | 141501 | 48006 | 138477 | 186482 | 48006 | 159563 | 207568 | 48006 | *nad5* exon1*, nad5* exon2*, trnC-*GCA*, trnN-*GTT*, trnY-*GTA*, nad2* exon3*, nad2* exon4*, nad2* exon5*, trnfM-*CAT*, rrn26, mttB, trnS-*GCT*, trnF-*GAA*, trnP-*TGG |
| Syntenic block5 | 141353 | 161783 | 20431 | 73476 | 93906 | 20431 | 207420 | 227850 | 20431 | *trnN-*GTT*, cob* |
| Syntenic block6 | 161363 | 178791 | 17429 | 26641 | 44072 | 17432 | 257247 | 274676 | 17430 | *rpl5, rps12, nad3* |
| Syntenic block7 | 187688 | 248171 | 60484 | 253578 | 314061 | 60484 | 356141 | 416624 | 60484 | *rrn5, rrn18, nad1* exon4*, matR, nad5* exon4*, nad5* exon5*, nad6, rps4, nad4* |
| Syntenic block8 | 248005 | 258289 | 10285 | 235135 | 245419 | 10285 | 346023 | 356307 | 10285 | *rpl2, rpl10, atp9* |
| Syntenic block9 | 258167 | 280331 | 22165 | 96728 | 118880 | 22153 | 323993 | 346145 | 22153 | *ccmFc, trnL-*CAA*, ccmC, rpl16, rps3, rps19* |
| Syntenic block10 | 283901 | 292878 | 8978 | 421349 | 430316 | 8968 | 1 | 9011 | 9011 | *atp6* |
| Syntenic block11 | 292729 | 308358 | 15630 | 430167 | 445793 | 15627 | 306884 | 322500 | 15617 | *cox2* exon3 |
| Syntenic block12 | 308819 | 314904 | 6086 | 221 | 6306 | 6086 | 300114 | 306199 | 6086 | *cox2* exon1*, cox2* exon2*, rpl16, rps3, rps19* |
| Syntenic block13 | 315455 | 334428 | 18974 | 6857 | 25831 | 18975 | 54538 | 73511 | 18974 | *trnQ-*TTG*, trnG-*GCC*, trnM-*CAT |
| Syntenic block14 | 334303 | 343612 | 9310 | 43652 | 53042 | 9391 | 248279 | 257667 | 9389 | *nad1* exon5*, nad4L, atp4* |
| Syntenic block15 | 343192 | 362794 | 19603 | 54280 | 73896 | 19617 | 227430 | 247006 | 19577 | *trnS-*TGA*, trnD-*GTC*, trnS-*GGA*, sdh4, cox3, atp8, rps1* |
| Syntenic block16 | 366815 | 378880 | 12066 | 223196 | 235257 | 12062 | 8190 | 20251 | 12062 | *trnI-*CAT |
| Syntenic block17 | 378072 | 383886 | 5815 | 218190 | 224004 | 5815 | 290671 | 296507 | 5837 | *sdh3, nad2* exon1*, nad2* exon2 |
| Syntenic block18 | 384265 | 392188 | 7924 | 209888 | 217811 | 7924 | 46628 | 54551 | 7924 | *trnW-*CCA*, trnP-*TGG*, nad9, trnH-*GTG |
| Syntenic block19 | 400564 | 421891 | 21328 | 187532 | 208855 | 21324 | 24272 | 45595 | 21324 | *trnK-*TTT*, ccmB, nad5 exon3, atp1* |

Start^a^ : Start position of syntenic block in mitogenome

End^b^ : End position of syntenic block in mitogenome

Length^c^ : Length of syntenic block in mitogenome
