# Supplementary material for: Organellar genome analysis reveals endosymbiotic gene transfers in tomato
Source: PLoS One. 2018 Sep 5;13(9):e0202279. doi: 10.1371/journal.pone.0202279 (PMC6124701; doi:10.1371/journal.pone.0202279)
Supplement: S4 Table — (DOCX) [file pone.0202279.s015.docx]

**S4 Table. Nuclear copies of plastid DNA (NUPTs) in the nuclear genomes of tomato species.**

| Comparison | Position | No. of NUPTs | Mean length (bp) | Median length (bp) | Maximum length (bp) | Minimum length (bp) |
| --- | --- | --- | --- | --- | --- | --- |
| plastome of *S. pennelli* ‘LA0716’  vs  nuclear genome of *S. pennelli* ‘LA0716’ | ch01 | 1156 (160)^a^ | 201.5 (788.7) | 109.5 (401) | 6536 | 35 (252) |
|  | ch02 | 550 (70) | 273.7 (1395.3) | 107.5 (342.5) | 13844 | 35 (250) |
|  | ch03 | 576 (68) | 138.6 (364.9) | 104 (304) | 1195 | 35 (250) |
|  | ch04 | 612 (92) | 204.1 (731.4) | 112 (342.5) | 8035 | 35 (251) |
|  | ch05 | 601 (86) | 154.1 (445.7) | 103 (356.5) | 2995 | 36 (250) |
|  | ch06 | 572 (74) | 189.2 (638.1) | 124 (329) | 9585 | 37 (251) |
|  | ch07 | 579 (75) | 191.5 (744.6) | 105 (390) | 9855 | 35 (253) |
|  | ch08 | 542 (84) | 230.3 (912.2) | 102 (368) | 13567 | 35 (256) |
|  | ch09 | 563 (73) | 179.5 (688.4) | 97 (358) | 5021 | 35 (255) |
|  | ch10 | 725 (171) | 453.4 (1568.9) | 120 (1005) | 9323 | 36 (251) |
|  | ch11 | 654 (108) | 213.7 (739.7) | 108 (388.5) | 10465 | 35 (253) |
|  | ch12 | 675 (122) | 215.1 (710) | 103 (390) | 5178 | 39 (250) |
| plastome of *S. lycopersicum* ‘LA1479’  vs  nuclear genome of *S. lycopersicum* ‘Heinz1706’ | ch01 | 1001 (143) | 273.8 (1284.3) | 103 (395) | 63335 | 35 (250) |
|  | ch02 | 478 (67) | 258.7 (1176.8) | 110 (348) | 10990 | 36 (257) |
|  | ch03 | 650 (116) | 216.7 (715.5) | 107.5 (388.5) | 3119 | 35 (252) |
|  | ch04 | 578 (81) | 204.7 (780.6) | 108 (411) | 4916 | 35 (252) |
|  | ch05 | 580 (81) | 150.1 (427) | 105 (355) | 1524 | 35 (250) |
|  | ch06 | 502 (55) | 152.8 (466.9) | 108 (329) | 2047 | 39 (250) |
|  | ch07 | 580 (71) | 172.4 (639.2) | 100 (394) | 5166 | 36 (252) |
|  | ch08 | 533 (74) | 174.3 (601.7) | 103 (359) | 6380 | 37 (252) |
|  | ch09 | 611 (78) | 181.9 (717.2) | 98 (362) | 7129 | 35 (252) |
|  | ch10 | 600 (83) | 184.8 (635) | 110 (375) | 3874 | 35 (251) |
|  | ch11 | 680 (104) | 225.1 (870.9) | 106.5 (368) | 10689 | 35 (250) |
|  | ch12 | 652 (124) | 222.7 (710.6) | 108 (389) | 5163 | 35 (252) |

^a^Numbers within parenthesis represent the respective values for NUPTs longer than 250 bp
